# Severe flooding and cause-specific hospitalisation among older adults in the USA: a retrospective matched cohort analysis

**DOI:** 10.1016/S2542-5196(25)00132-9

**Published:** 2025-07-30

**Authors:** Sarika Aggarwal, Jie K Hu, Jonathan A Sullivan, Robbie M Parks, Rachel C Nethery

**Affiliations:** Department of Biostatistics, Harvard T H Chan School of Public Health, Boston, MA, USA; Department of Statistics, The Ohio State University, Columbus, OH, USA; Nelson Institute for Environmental Studies, University of Wisconsin–Madison, Madison, WI, USA; Department of Environmental Health Sciences, Columbia University Mailman School of Public Health, New York, NY, USA; Department of Biostatistics, Harvard T H Chan School of Public Health, Boston, MA, USA

## Abstract

**Background:**

Floods are the most common climate-related disaster; yet previous studies have investigated the impact of floods on only a few health outcomes in narrow spatiotemporal settings. We aimed to assess the association between severe flood exposure and cause-specific hospitalisation rates in adults older than 65 years in the contiguous USA.

**Methods:**

In this retrospective matched cohort analysis, we obtained inpatient claims data from Medicare fee-for-service beneficiaries older than 65 years living in the contiguous USA from Jan 1, 2000, to Dec 31, 2016. From each inpatient hospitalisation record, we extracted the admission date, primary International Classification of Diseases, 9th revision, clinical modification (ICD-9-CM) code (or 10th revision [ICD-10-CM] code on or after Oct 1, 2015), and self-reported residential ZIP code. Hospitalisation data were linked with satellite-based, high-resolution historical flood maps from the Global Flood Database by ZIP code. Days during and shortly after a flood exposure were matched to non-flood-affected control days by ZIP code and day-of-year. We estimated relative percentage changes in hospitalisation rates for 13 mutually exclusive, well-defined disease categories during and in the 4 weeks following flood exposure with conditional quasi-Poisson regression models.

**Findings:**

This study captured 72 major flood events and included over 4·5 million hospitalisations occurring over a 17-year period. We observed elevated rates of hospitalisation on average during and following flood exposure for skin diseases (3·1% [95% CI 1·4 to 4·9]), nervous system diseases (2·5% [1·0 to 4·1]), musculoskeletal system diseases (1·3% [0·3 to 2·3]), and injuries or poisoning (1·1% [0·2 to 2·0]). Communities with lower proportions of Black residents experienced exacerbated flood effects for nervous system diseases (7·6% [95% CI 2·8 to 12·6]), whereas skin diseases (6·1% [1·9 to 10·5]) and mental health-related impacts (3·0% [−0·3 to 6·5]) were more pronounced for areas with larger percentages of Black residents during flood exposure.

**Interpretation:**

Among adults older than 65 years, exposure to severe flood events was associated with increased hospitalisation rates for skin diseases, nervous system diseases, musculoskeletal system diseases, and injuries. Different patterns of hospital admission persisted for populations with higher versus lower proportions of Black residents. Our findings indicate a need for targeted flood-specific preparedness and adaptation strategies for socially vulnerable populations, including older individuals and racially minoritised communities.

## Introduction

Since 2000, flooding has been the most frequently occurring climate-related hazard,^1,2^ and increases in frequency and intensity are projected in the coming decades due to climate change, urbanisation, and increasing settlement in floodplains.^3–5^ In the USA, socially vulnerable populations (such as older adults and racially minoritised and low-income groups) disproportionately reside in flood zones.^6–9^ Older adults are particularly vulnerable to flood-related health impacts because of their weakened immune systems, restricted mobility, limited ability to cope with climate hazards due to pre-existing conditions such as dementia, and poor access to medical services for routine appointments.^10–13^

Previous epidemiological studies, which have largely been limited to localised case studies investigating a small number of health outcomes,^2^ have observed flood-related increases in risk of injuries,^14,15^ gastrointestinal and skin infections (mostly in low-income countries),^16–19^ cardiovascular illness,^20^ and mental health problems.^21^ However, given the diverse pathways through which floods can influence health and wellbeing,^2^ the scope of health impacts is probably broader than currently recognised. This knowledge gap impedes informed strategic preparedness efforts that could minimise the adverse health impacts of floods.

In this study, we aimed to combine population-level Medicare claims data and high-resolution satellite-based flood exposure measures to examine how flood exposure is associated with 13 diverse categories of cause-specific hospitalisation in adults older than 65 years over a 17-year study period in the contiguous USA. We investigated whether and how the rates of each type of hospitalisation change in flood-exposed ZIP codes both during and for 1 month following exposure relative to a business-as-usual scenario. This approach captures wide-reaching direct health impacts (ie, events causally linked to floods) and indirect health impacts (ie, events not individually attributable to a flood but for which floods modify population-level risks) occurring in the immediate and medium-term aftermath of flood exposures. Moreover, we aimed to investigate whether marginalised communities experience differential flood-related health impacts.

## Methods

### Study design and population

We conducted a retrospective matched cohort analysis with Medicare inpatient claims data provided by the US Centers for Medicare and Medicaid Service. Medicare is a federal health insurance programme available to American citizens older than 65 years. The data used in this study contain demographic information, self-reported ZIP codes of residence (ie, billing ZIP codes), and records of each inpatient hospitalisation for all Medicare fee-for-service beneficiaries who were enrolled for at least 1 month from Jan 1, 2000, to Dec 31, 2016, and living in the contiguous USA (appendix p 2). From each hospitalisation record, we extracted the date of admission and primary International Classification of Diseases, 9th revision, clinical modification (ICD-9-CM) code (or 10th revision [ICD-10-CM] code on or after Oct 1, 2015). This study was approved by the Harvard Longwood Campus Institutional Review Board, IRB20–1910 Tropical Cyclones.

### Procedures

We grouped ICD-9-CM and ICD-10-CM codes with the clinical classifications software (CCS) algorithm into 18 mutually exclusive, clinically meaningful CCS level 1 causes of hospitalisation.^22^ We excluded five causes of hospitalisation that are ill-defined or not relevant to the Medicare cohort (eg, pregnancy; appendix p 2) and investigated the remaining 13 level 1 causes: infectious and parasitic diseases; neoplasms; endocrine system disorders; blood diseases; nervous system diseases; circulatory system diseases; respiratory system diseases; digestive system diseases; genitourinary system diseases; skin and subcutaneous tissue diseases; musculoskeletal system diseases; injury and poisoning; and mental illness. ZIP code-level counts (and rates) of hospitalisation for each cause were calculated according to the category corresponding to the primary diagnosis code for each admission.

We obtained data on severe flood exposures that occurred in the contiguous USA from 2000 to 2016 with the Global Flood Database, the most comprehensive collection of satellite-based high-resolution historic flood maps to date.^23^ The creation of these flood maps is described in the appendix (p 2). Each flood event was given one of three severity labels by the Dartmouth Flood Observatory (adopted by the Global Flood Database)^24^ depending on the scale of the event and the expected recurrence interval, which we refer to as moderate, high, and extreme severity (appendix p 2). For our primary analysis, we only considered floods caused by heavy rain or tropical storms.

To link flood exposure information with our health records, we aggregated the flood data to the ZIP code level, which is the finest geographical resolution at which residential location is recorded in Medicare claims. For our main analysis, we classified a given ZIP code and day as being exposed if a mapped flood event revealed flooding of at least 0·5% or flooding of at least 5 square miles of the ZIP code’s surface area.

To adjust for time-varying covariates, we obtained daily 4-km x 4-km gridded maximum air temperature, maximum relative humidity, and wind velocity data from the Gridded Surface Meteorological Dataset. We also used daily 1-km x 1-km gridded ambient fine particulate matter (PM_2·5_), ozone (O_3_), and nitrogen dioxide (NO_2_) pollutant concentrations from the NASA Socioeconomic Data and Applications Center. Gridded measures were aggregated to the ZIP code level.^25^ Additionally, we collected information on ZIP code-level socioeconomic status indicators, including the proportion of Black residents, from the US Census Bureau data^26^ to examine health inequities in stratified analyses.

### Statistical analysis

Following previous work,^27^ we used a self-matched design combined with a conditional quasi-Poisson modelling approach to assess the change in rate of each cause of hospitalisation relative to a business-as-usual scenario during flood exposures and the subsequent 4 weeks (appendix p 3). We matched each flood exposure period (ie, a set of consecutive flooded days) for a given ZIP code to two non-flooded control periods in the same ZIP code and days of the year but in years preceding or following the particular flood exposure. Our analytical dataset was composed of rates of each cause of hospitalisation during the flood exposure periods, the matched control periods, and each of the 4 lag weeks following each exposure and control period. We included 4 lag weeks in the analyses to investigate any potentially delayed, medium-term health effects. We applied conditional quasi-Poisson distributed lag models to each cause of hospitalisation separately. We adjusted for potential confounders that were not accounted for through matching (such as long-term trends in hospitalisation patterns and day-to-day meteorology) by including natural splines with two degrees of freedom for the year and means of air temperature, relative humidity, windspeed, and each pollutant (ie, PM_2·5_, NO_2_, and O_3_). We report estimated relative percentage changes ([relative risk – 1] × 100) in hospitalisation rates during each flood exposure period and lag week. To address multiple comparisons, we used the Bonferroni method to correct 95% CIs.

We additionally investigated whether estimated effects varied by season (cold *vs* warm), flood severity (moderate *vs* high or extreme), or admission type (emergency *vs* non-emergency) with stratified analyses. We also used stratified analyses to examine how effects differed for marginalised communities, specifically for ZIP codes with higher versus lower proportions of Black residents, given the disproportionate increase in flood exposure risk predicted for Black communities,^8^ and ZIP codes with higher versus lower socioeconomic status (defined by median household income or poverty rate).

We investigated associations between flood exposure and individual subcauses of hospitalisation that accounted for 20000 or more hospitalisations in our study sample (appendix p 5). To further characterise medium-term impacts of flood exposure, we used an extended lag period of 12 weeks in a secondary analysis. We conducted sensitivity analyses, considering several alternative flood exposure definitions and different approaches to confounding adjustment. More details are provided in the appendix (pp 3–4).

### Role of the funding source

The funders of the study had no role in study design, data collection, data analysis, data interpretation, or writing of the report.

## Results

**Figure 1:**
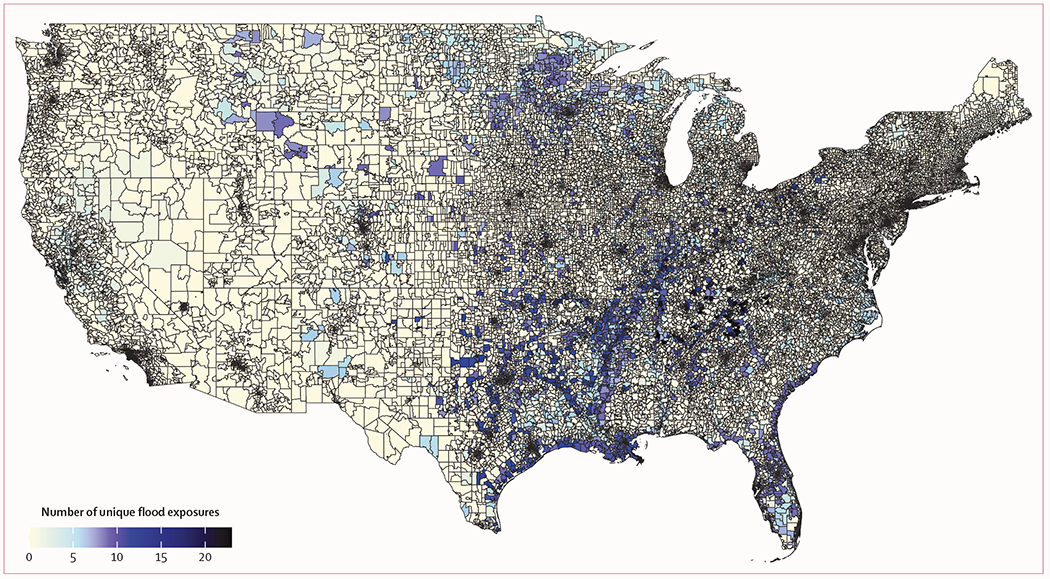
Spatial trends in flood exposure across ZIP codes in the contiguous USA from 2000 to 2016

Our study included 72 distinct flood events during 2000–16 that ranged in duration from 1 day to 42 days. Of these 72 events, 40 (56%) were of moderate severity, ten (14%) were of high severity, and 22 (30%) were of extreme severity. Heavy rain was the primary cause for 58 (81%) of the 72 flood events with the remaining 14 (19%) a result of tropical storms and surges. These flood events covered 11562 unique ZIP codes in 46 states and the District of Columbia (figure 1). Flood-affected areas ranged from 56·75 square miles to 22819·72 square miles, with a median of 806·95 (IQR 360·38–1652·55) square miles. Flood exposures were most common in the Mississippi and Ohio River valleys and along the Gulf and southeastern Atlantic coasts. Temporal trends in the number of Medicare enrollees living in flood-exposed ZIP codes over the study period are shown in the appendix (p 11).

There were 4878158 recorded hospitalisations among Medicare enrollees in the ZIP codes and days included in our analytical dataset. Diseases of the circulatory system (1530770 [31·4%]), respiratory system (679997 [13·9%]), and digestive system (526512 [10·8%]), and injury and poisoning (466 745 [9·6%]) were the leading causes of hospitalisation. Crude rate ratios of hospitalisation sorted by cause during flood exposure (ie, lag 0) and in each of the 4 weeks following exposure (ie, lag weeks 1–4), relative to the matched controls, are shown in the appendix (p 12).

**Figure 2:**
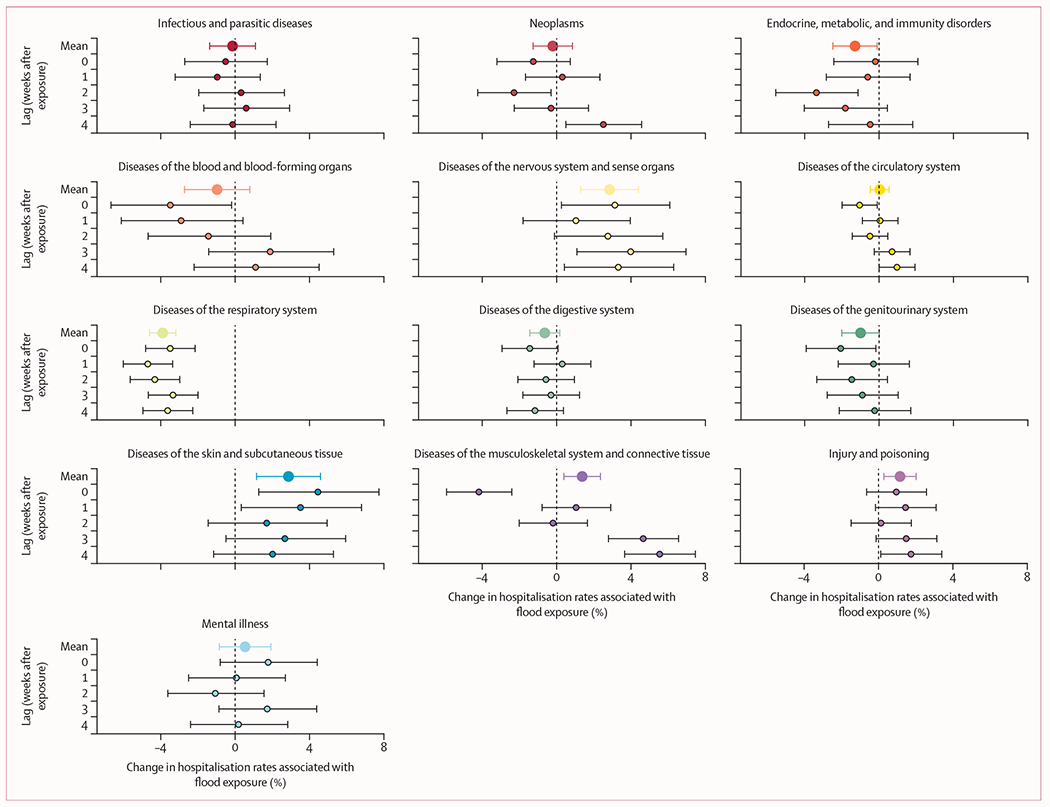
Percentage changes in cause-specific hospitalisation rates during and after flood exposure by cause and lag time Dots show point estimates and error bars represent Bonferroni-corrected 95% CIs. The mean percentage change in hospitalisation (with corresponding corrected 95% CIs) over the flood exposure period and all 4 lag weeks is shown at the top of each panel.

The estimated relative percentage change in rates of hospitalisation during flood exposure (ie, lag 0) and each of the 4 following weeks (ie, lags 1–4) and the mean percentage changes across the studied time periods are shown in figure 2. Significant increases in hospitalisation rates, on average, across the exposure and lag weeks were detected for skin diseases (3·1% [95% CI 1·4 to 4·9]), nervous system diseases (2·5% [1·0 to 4·1]), musculoskeletal system diseases (1·3% [0·3 to 2·3]), and injuries or poisoning (1·1% [0·2 to 2·0]). We observed the largest increase in hospitalisation rates for diseases of the musculoskeletal system in the fourth week after flood exposure (5·6% [95% CI 3·7 to 7·6]). Risks of hospitalisation due to nervous system diseases were elevated during flood exposure and each of the 4 weeks after flooding, with a maximum risk in the third week following exposure (4·0% [95% CI 1·1 to 7·0]). Hospital admissions for diseases of the skin were similarly elevated during exposure and all 4 lag weeks, but with a peak increase during flood exposure (4·5% [95% CI 1·3 to 7·7]). We also observed elevated risk for injury and poisoning across the exposure and lag periods, with a peak increase of 1·7% (95% CI 0·1 to 3·4). Conversely, we observed the largest decrease in hospitalisation rates for respiratory diseases (−4·7% [95% CI −6·0 to −3·4]) during the first week after flood exposure. Diseases of the respiratory system were the only cause of hospitalisation that showed flood-driven decreases across the exposure period and each lag week (figure 2).

There was an increased risk for circulatory system diseases and neoplasms in lag week 4 following exposure (figure 2). For mental illness, the digestive system, and infectious and parasitic diseases, we did not observe noteworthy deviations from expected hospitalisation rates across the studied time periods (figure 2). There was a significant decrease in diseases of the blood (−3·5% [95% CI −6·7 to −0·2]) and genitourinary system hospitalisations (−2·1% [−3·9 to −0·2]) during flood exposure and in endocrine system hospitalisations (−3·4% [−5·6 to −1·1]) during the second week after flood exposure.

**Figure 3:**
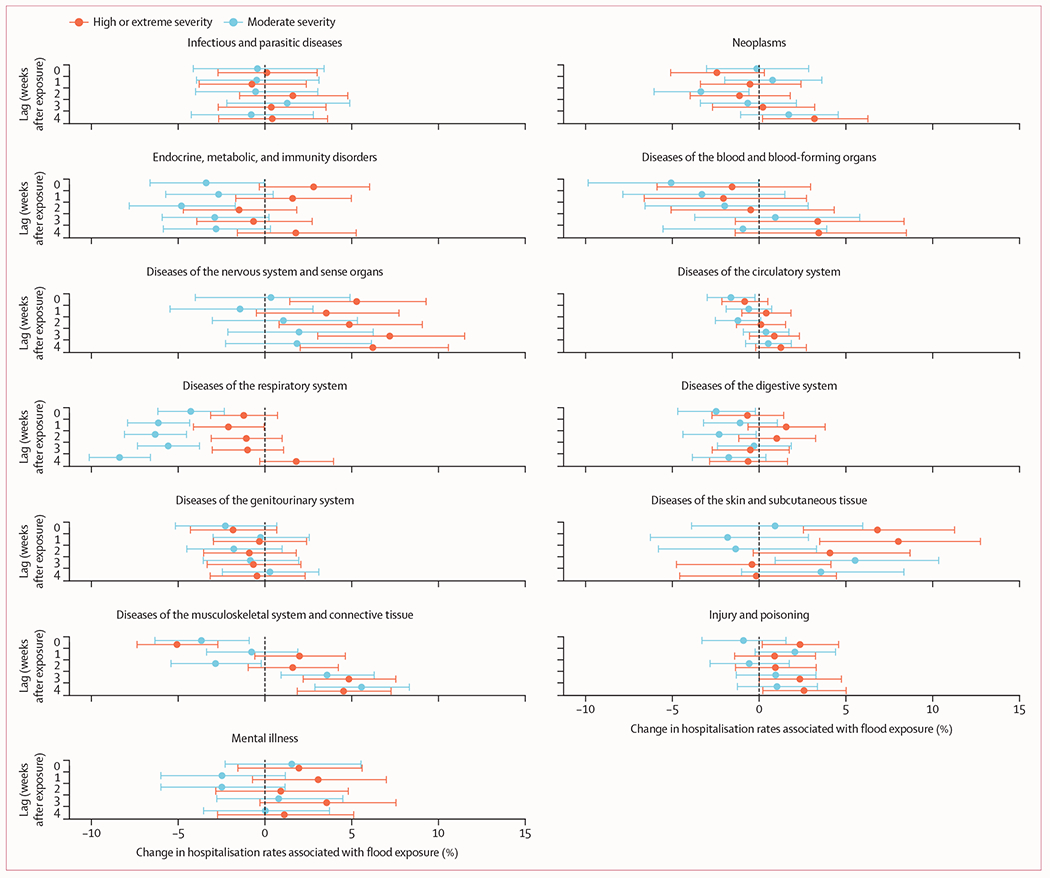
Severity-stratified percentage changes in cause-specific hospitalisation rates during and after flood exposure by cause and lag time Dots show point estimates and error bars represent Bonferroni-corrected 95% CIs.

In models stratified by flood severity, we generally found larger and more consistent adverse health impacts of high-severity or extreme-severity floods relative to those of moderate severity (figure 3) across causes and lag periods. These exacerbated effects of more severe floods were especially pronounced for diseases of the nervous system and mental illness across the exposure period and lag weeks, and for skin diseases during the exposure period and the first two lag weeks. We also observed substantial differences in flood-related changes in respiratory disease hospitalisations for moderate-severity versus high-severity or extreme-severity floods, especially during lag week 4. The results stratified by season and type of admission are presented in the appendix (pp 7–8).

**Figure 4:**
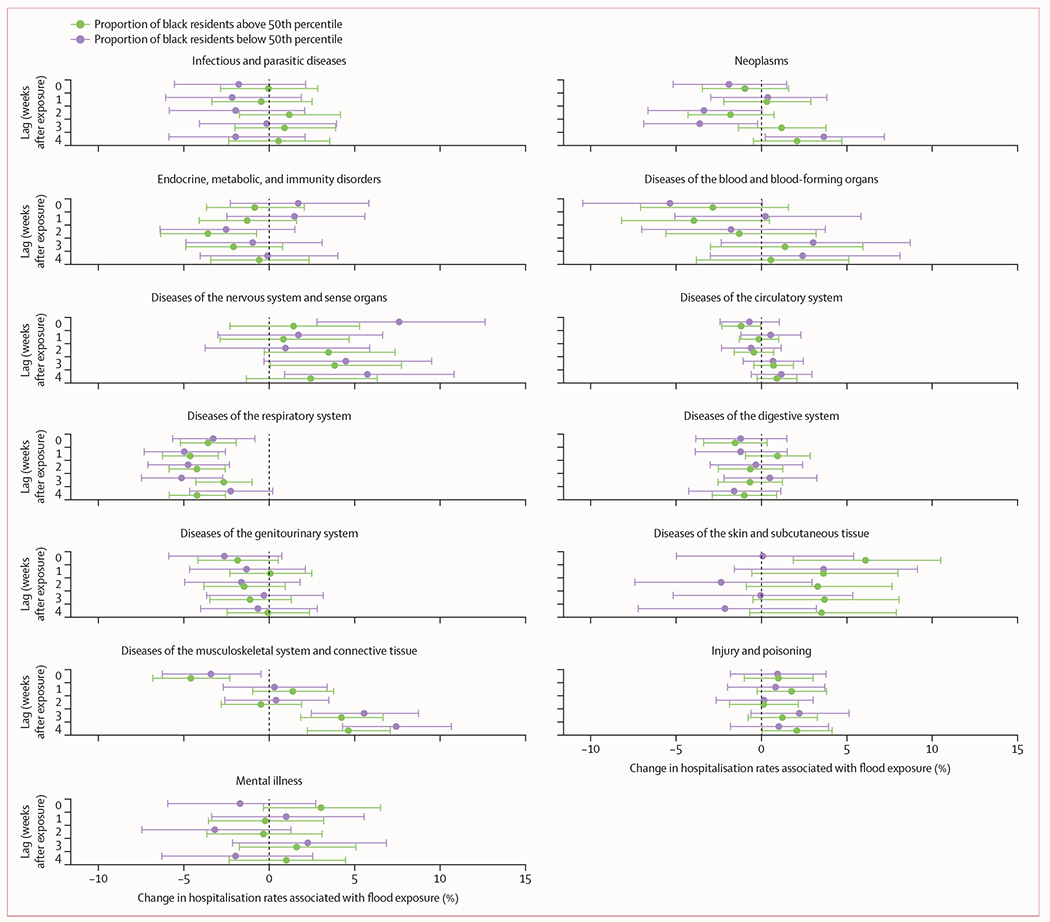
Percentage changes in cause-specific hospitalisation rates during and after flood exposure, by cause and lag time, stratified by ZIP code-level proportion of Black residents Dots show point estimates and error bars represent Bonferroni-corrected 95% CIs.

Figure 4 shows the results for models stratified by the percentage of residents in each ZIP code who identify as Black. For nervous system diseases, we observed exacerbated flood effects for communities with lower percentages of Black residents across most lag periods, particularly during flood exposure (7·6% [95% CI 2·8 to 12·6]). These communities also had larger estimated effects during lag weeks 3–4 for diseases of the musculoskeletal system and connective tissues (figure 4). However, we observed larger flood-related increases in hospitalisation rates for skin diseases (6·1% [95% CI 1·9 to 10·5]) for ZIP codes with higher proportions of Black residents than those with lower proportions of Black residents during the exposure period and most lag weeks (figure 4). ZIP codes with a higher percentage of Black residents also showed an increase in hospitalisations for mental illness during flood exposure (3·0% [95% CI −0·3 to 6·5]), whereas ZIP codes with a lower percentage of Black residents did not. Model results stratified by ZIP code-level median household income and poverty rate are provided in the appendix (pp 9–10).

In our secondary analysis with an extended lag period of 12 weeks, we observed increased rates of hospitalisation for respiratory system diseases during the later lag weeks (ie, weeks 8–12) with a peak increase during lag week 10 (4·1% [95% CI 2·7–5·5]). Other causes of hospitalisation exhibited patterns consistent with the primary analysis (appendix p 13).

In our sensitivity analyses that considered different flood exposure definitions, we found similar results with only minor changes in magnitude (appendix pp 14–17). Our results were also robust to different confounding adjustment procedures (appendix pp 18–22). Results of the subcause analysis are provided in the appendix (p 6).

## Discussion

We used nationwide Medicare data for a 17-year period and satellite-based flood maps to conduct the first large-scale study of how flood exposure is associated with rates of hospitalisation in older adults for 13 disease causes. We observed that flood exposure was associated with increases in hospitalisation rates for skin diseases, nervous system diseases, musculoskeletal system diseases, and injuries. Patterns of association across lag weeks varied between acute and chronic causes, which might be due to initial disruptions in access to health care or longer-term exacerbation of chronic conditions. Exposure to higher-severity floods amplified adverse impacts. The association between floods and hospitalisation rates revealed differing trends in populations with higher versus lower proportions of Black residents.

Previous studies on the association between flood exposure and health outcomes have primarily investigated only a single flood event or a specific geographical region. Following floods from Hurricane Katrina, authors observed increased odds for calls related to injury and poisoning in the first 12 weeks post flood,^28^ increased odds of skin rashes,^29^ cold and influenza-like symptoms,^30^ and outbreaks of viral gastroenteritis for 4% of evacuees in a mega shelter in the first 4 weeks post flood.^31^ Previous investigations on psychosocial conditions have identified long-term impacts of increased post-traumatic stress disorder, anxiety, and depression;^13,16,18,32–34^ however, evidence is contradictory for older adults.^35^ Studies have also observed increased risks of all-cause mortality in the UK (6·7% [95% CI 6·3–7·1])^36^ and worldwide (2·1% [0·6–3·6]),^37^ and increased risk of cause-specific mortality in the USA^38^ following flood exposure.

We observed increased hospitalisation rates during and after flood exposure for diseases of the nervous system. Acute, provoked seizures and long-term post-traumatic epilepsy could arise from traumatic brain injuries sustained during floods.^39^ Our subcause analysis (appendix p 6) provides empirical support for this mechanism. For individuals with epilepsy, flood events can induce stress, fatigue, and sleep deprivation, which might result in poor seizure control and other epilepsy-related symptoms.^40^ Headaches, conjunctivitis, and otitis media can also occur due to the presence of contaminated floodwater.^41–45^

The observed elevated rates of skin and subcutaneous tissue diseases during and after flood exposure are plausible as floods can result in polluted and contaminated water sources, crowded shelters, and poor sanitation.^46,47^ Exposure to stagnant water can lead to cutaneous and fungal infections, infections of traumatic wounds, and insect bites.^48–50^ Our findings agree with previous reports of increased skin diseases from studies of single-event catastrophic flood disasters in the USA and globally.^46,48,51^ Literature suggests that the 28 days following a flood represent the critical periods of dermatological risk, which is consistent with our findings of elevated skin disease hospitalisations for several weeks post flood.^51^

Because a substantial proportion of musculoskeletal system hospitalisations are for chronic subcauses, individuals might delay seeking care during a flood due to risks associated with traveling to a hospital.^52^ This delay might lead to increased hospitalisation rates in subsequent weeks, consistent with what we observed, as patients reschedule care that was disrupted. Furthermore, flood clean-up requires repetitive, unfamiliar movements such as bending and lifting heavy weights, which might strain muscles or joints and aggravate existing chronic issues.^53,54^

We observed an elevated risk of hospitalisation for injuries and poisoning during and in the weeks following flood exposure. Injuries can arise from unsafe conditions during a flood, evacuation attempts, clean-up or repairs following a flood, generator use, or the operation of vehicles in floodwater.^14,42,55,56^ In previous floods in the American midwest, reported hospitalisations largely included sprains and strains, contusions, and lacerations,^14^ coinciding with our subcause analysis (appendix p 6).

We observed a decreased risk of respiratory hospitalisations during flood exposure and lag weeks 1–4, but an increased risk during the 8–12 weeks post flood in our secondary analysis, indicating that adverse respiratory impacts might materialise several months after exposure. Studies of hurricanes have identified short-term increases in respiratory risks,^27,57,58^ largely hypothesised as being driven by power outages caused by strong winds (which disrupt the use of electric-powered breathing equipment). However, heavy precipitation events that lead to flooding are not always accompanied by high winds. Inverse associations between floods and respiratory hospitalisations were driven by less severe floods, which might result in minimal flooding of residential areas. Any associated heavy precipitation could be protective for short-term respiratory health because rainfall settles dust^59^ (a respiratory irritant), whereas long-term adverse associations could occur due to indoor dampness and mould exposure.^60^

Previous literature suggests that standing water from flood events can lead to the spread of waterborne diseases and a subsequent increase in infectious, parasitic, or digestive system diseases.^14,46,47,61^ However, most of the empirical evidence of these findings is from low-income countries and covers outcomes less severe than inpatient hospitalisation.^16^ Effects for these disease types were not observed in our inpatient hospitalisation study in a high-income country.

Some socially vulnerable groups, such as low-income and minoritised individuals, disproportionately reside in flood-prone areas in the USA.^7,9,62–64^ This disproportionate exposure burden is predicted to persist and widen for Black communities in the coming years due to climate change.^8,65,66^ Beyond unequal exposure risks, we identified differences in flood-related health outcomes for communities with larger versus smaller proportions of Black residents. Distinct patterns of pre-existing conditions, housing quality, and access to emergency resources suggest that some health impacts of flooding might be more acute for racially marginalised groups. However, another possible explanation for observed differences in health impacts is differences in access to care and implicit biases in the coding of disease conditions between racial groups. For instance, we observed increases in nervous system hospitalisations in communities with lower proportions of Black residents and increases in mental illness-related hospitalisations in communities with higher proportions of Black residents. These results could indicate differences in access to neurological care across communities or differences in how similar conditions are coded across groups.^67,68^ Flood-driven increases in skin disease hospitalisations were also more prominent in communities with larger Black populations. Racially minoritised populations have been found to experience greater incidence of skin disease,^69^ which might be exacerbated by climate-change-driven disasters.

Although this study is novel in characterising the far-reaching health implications of flood events, this work has limitations. The Global Flood Database does not capture all severe flood events, as many floods cannot be mapped via satellite imaging due to persistent cloud cover or their fast-moving nature. However, our self-matched study design compares health outcomes in areas identified as experiencing flood exposure with those same areas at times without exposure, allowing us to remain agnostic about the exposure status of areas where floods were not mapped in the Global Flood Database. Although flooding tends to be a highly localised event, we summarised flood exposures at the ZIP code level for linkage with Medicare records. Thus, not everyone living in an exposed ZIP code necessarily experienced residential flooding, making exposure misclassification plausible. However, flood events can affect the health of even community members whose homes are not flooded, and our approach allows us to capture any such effects, including changes that result from disruptions to health-system functioning and access to health care. We found that our results were consistent across different exposure definitions. Each individual ICD-9-CM or ICD-10-CM code might not capture all nuances of disease causes; however, our outcomes are coarse groupings of these diagnosis codes, which reflect well-defined, broad causes of hospitalisation. As in any observational study, residual confounding cannot be entirely ruled out. However, we leveraged a robust matched study design that controls for time-invariant factors across ZIP codes and seasonality while additionally adjusting for longer-term time trends, meteorology, and air pollution.

In conclusion, flood exposure was associated with increased rates of hospitalisation for skin diseases, nervous system diseases, musculoskeletal system diseases, and injuries on average during and in the aftermath of flood exposure among adults older than 65 years. Higher-severity floods exacerbated adverse health effects. Additionally, patterns of association differed by community racial composition. Our findings might lend insights to ongoing efforts to develop protocols for building health-care system resilience and minimising the public health burden of severe flood events. Targeted outreach and robust evacuation planning for vulnerable populations, such as older individuals, along with community-based alert systems are crucial to minimising health impacts. Hospital infrastructure should be equipped to function during flood events by moving essential components above flood levels, and mobile medicine units and telemedicine can serve as effective alternatives if access to hospitals is temporarily eliminated. Drones can also deliver essential medical supplies to flood-affected hospitals or help identify safe evacuation routes in real time to guide emergency responders.^70–72^

## Supplementary Material

1

## Data Availability

Flood data are available via the Harvard Dataverse. Medicare enrollee data are publicly accessible (upon purchase after an application process) from the US Centers for Medicare and Medicaid Services. Code for analyses and data visualisations presented in this Article is available online.

## Research in context

### Evidence before this study

We searched Embase, PubMed, and Google Scholar to identify original studies published in English investigating flood events and health from database inception to May 31, 2024. We used the terms flood* or flooding* and searched for associations with adverse health outcomes with the terms disease* or illness* or hospitalization* or admission* or inpatient* or emergency department* or ED*. Existing evidence from global studies generally supports increased risk of communicable diseases (eg, infectious, digestive, or skin diseases) and mental health conditions after flooding events. However, most studies investigated the effects of only a single flood event on a limited set of health outcomes with little information on older individuals, used inconsistent exposure definitions, and did not include baseline measures or control groups. As such, current knowledge surrounding the health effects of floods is insufficient to inform strategic preparation and adaptation measures. New research is needed that empirically assesses the effect of flood exposure on hospitalisation risks for a broad spectrum of diseases in older adults with a nationwide, multidecade study design that leverages standardised flood exposure and outcome measures and a rigorous, unified analytical approach.

### Added value of this study

This study addresses the gaps identified above by analysing linked Medicare claims and satellite flood maps from Jan 1, 2000, to Dec 31, 2016. Among American individuals older than 65 years, floods were associated with increased risks of hospitalisation for diverse disease types, and different patterns emerged for racially minoritised populations. On average, we observed elevated rates of hospitalisation for skin, nervous system, and musculoskeletal system diseases and injuries during flood exposure and in the subsequent 4 weeks. Flood effects on nervous system diseases were more pronounced in areas with lower proportions of Black residents, whereas skin diseases and mental health-related impacts were exacerbated for communities with higher proportions of Black residents during flood exposures. This study fills an important research gap by identifying community-level effects of floods on disease causes and exploring associations for socially vulnerable populations. By analysing 72 floods and over 4·5 million hospitalisations across the contiguous USA for a 17-year period, our study provides robust, generalisable findings that can be used to inform flood preparation in high-income countries.

### Implications of all the available evidence

The findings of this study provide crucial new insights into the diverse, and previously underappreciated, health consequences of floods in older adults and can guide flood-specific resilience-building efforts to protect public health under climate change. Our identification of vulnerable subpopulations, including older adults and racially minoritised individuals, can inform targeted interventions to maximise the health benefits of strategic preparation in realistic resource-limited settings.
