# Supplementary material for: Severe flooding and cause-specific hospitalisation among older adults in the USA: a retrospective matched cohort analysis
Source: Lancet Planet Health. Author manuscript; Available in PMC 2025 Aug 18. (PMC12360453; doi:10.1016/S2542-5196(25)00132-9)
Supplement: 1 [file NIHMS2102289-supplement-1.pdf]

THE LANCET
Planetary Health

Supplementary appendix

This appendix formed part of the original submission and has been peer reviewed.
We post it as supplied by the authors.

Supplement to: Aggarwal S, Hu JK, Sullivan JA, et al. Severe flooding and cause-specific hospitalisation among older adults in the USA: a retrospective matched cohort analysis. *Lancet Planet Health* 2025. https://doi.org/10.1016/S2542-5196(25)00132-9

Table of Contents

S.1 Medicare data details . . . 2
S.2 Supplemental exposure assessment information . . . 2
S.2.1 Flood map creation . . . 2
S.2.2 Severity categorization . . . 2
S.3 Supplemental methods . . . 3
S.3.1 Statistical model . . . 3
S.3.2 Estimation of flood-related risks . . . 3
S.3.3 Multiple comparisons correction . . . 3
S.3.4 Additional analyses . . . 3
S.4 Sub-cause analysis . . . 5
S.5 Stratified analysis by season . . . 7
S.6 Stratified analysis by admission type . . . 8
S.7 Stratified analyses by socioeconomic status indicators . . . 9
S.8 Additional analyses . . . 11
S.8.1 Descriptive plots . . . 11
S.8.2 Evaluation of longer lag periods to further characterize medium-term impacts . . . 13
S.8.3 Sensitivity analyses: alternative flood exposure definitions . . . 14
S.8.4 Sensitivity analyses: residual confounding . . . 18

ICD-9-CM and ICD-10-CM codes

A table of ICD-9-CM and ICD-10-CM codes per CCS level 1 hospitalization causes used in analyses is available upon request.

S.1 Medicare data details

We used the Medicare Provider Analysis and Review (MEDPAR) data from the U.S. Centers for Medicare & Medicaid Services (CMS) to identify Medicare fee-for-service beneficiaries who were aged 65+, living in the contiguous USA, and had at least one hospitalization for a medical condition classified within the Clinical Classifications Software (CCS) level 1 defined disease groups during the period January 1, 2000 to December 31, 2016. We excluded five disease categories that are ill-defined or not relevant to the Medicare cohort: pregnancy and childbirth complications, congenital anomalies, conditions originating in the perinatal period, residual/unclassified codes, and symptoms; signs; ill-defined conditions and factors influencing health status. The MEDPAR data includes admission and discharge dates, ICD-9-CM/ICD-10-CM diagnosis codes, and ZIP code of residence for each Medicare fee-for-service beneficiary who was hospitalized in the USA. We constructed daily hospitalization counts for each ZIP code, i.e., the count of hospitalizations for each disease cause on a particular day and ZIP code. Then, we aggregated the daily hospitalization counts for each disease cause by ZIP code for a given flood event to create 5 distinct time periods: (1) the set of consecutive flooded days and (2-5) four subsequent lag weeks. We constructed analogously temporally-aggregated hospitalization counts for the matched control periods for each ZIP code-flood exposure (see Section S.3.1 for details about the matching procedure).

We used the annual Medicare beneficiary denominator file from CMS to identify all Medicare beneficiaries aged 65+ who were enrolled in the fee-for-fervice program between January 2000 and December 2016. The denominator file includes demographic information. From the denominator file, we calculated the number of Medicare fee-for-service beneficiaries who were at risk for hospitalizations by year and residential ZIP code.

The final analytic sample included the number of Medicare fee-for-service beneficiaries who were at risk and the number of hospitalizations that occurred for each of the 13 relevant CCS level 1 defined disease categories for each ZIP code-flood exposure period, each of the four lag weeks, and the corresponding matched control periods.

S.2 Supplemental exposure assessment information

S.2.1 Flood map creation

The creation of these flood maps was described in detail by [1], and we summarize key points and features here. To construct the flood maps, dates and approximate locations of major flood events were identified using the Dartmouth Flood Observatory event catalogue [2]. The Dartmouth Flood Observatory includes more flood events than other listings such as the Emergency Events Database (Em-Dat) [3], identifies the proposed cause of the flood (heavy rain, tropical storm, snow/ice melt, or dam), and provides critical spatial information that can be used to leverage flood detection algorithms on satellite imagery.

High-resolution flood maps were created for a selection of events in the Dartmouth Flood Observatory catalogue where satellite imagery was capable of producing inundation footprints with high accuracy. Flood maps were generated by analyzing daily satellite imagery from NASA's Moderate Resolution Imaging Spectroradiometer (MODIS) satellites at 250-meter resolution over the relevant area and time period [4]. To do this, the following steps were taken. First, the authors defined the region of interest to map for the potential flood event by selecting all global HydroSHEDS watersheds that intersect with the Dartmouth Flood Observatory event polygon. Then they aligned resolution of reflectance values in visible, near-infrared, and short-wave-infrared wavelengths using a corrected reflectance algorithm. Next, an existing algorithm was deployed on satellite-measured reflectance to classify each pixel in the region of interest as surface water or non-surface water. Quality control measures were undertaken after, e.g., to reduce misclassification from other weather elements such as cloud shadows, and accuracy was assessed.

S.2.2 Severity categorization

As defined by the Dartmouth Flood Observatory and adopted by the Global Flood Database, flood severity class takes on 3 distinct values: 1, 1.5, or 2 which we describe as "moderate", "high" and "extreme", respectively [2]. Severity class is determined based on the scale of the event and expected recurrence intervals using historical data.

Namely, floods of class moderate are large events with substantial damage/fatalities that have a 10-20% chance of occurring in a single year. Floods of class high are larger with recurrence interval of 21-99 years and/or locally recurring within 1-2 decades and affecting a significantly large geographic region. Floods of class extreme are the most severe, with recurrence greater than 10 decades [2].

S.3 Supplemental methods

S.3.1 Statistical model

For each flood event, we first determined each ZIP code's exposure period (the set of days when that particular ZIP code was flooded). Then, we matched each ZIP code-flood exposure period to two control periods from the same ZIP code and the same time period (i.e. days-of-year) but in years preceding or following flood exposure to adjust for bidirectional time trends. Matching time periods were selected on the basis of the closest two years with no flood exposure during the equivalent days-of-year or lag time (four subsequent weeks). These control periods allowed us to establish a baseline rate of hospitalization (absent flood) in each ZIP code under study. With this study design, comparisons between the flooded ZIP codes and matched controls address both time-invariant ZIP code features (by matching on ZIP code) and seasonal trends in the outcome (by matching on day-of-year) that may otherwise bias results. We defined a "stratum" as a given ZIP code-exposure period, its matched control periods, and all of the lag weeks following each flooded and control period (four lag weeks were used in the main analysis). ZIP code hospitalization counts were aggregated over each exposure/control period and each lag week separately. Our analytic sample was composed of the data for all strata across the 72 flood events.

We index strata by s and each of the units/periods within strata by t. The model takes the following form:

$$\log\left(E[Y_{st}]\right) = \alpha_0 + \alpha_s + \sum_{\ell=0}^{4} \beta_\ell \text{Exposure}_{\ell st} + \boldsymbol{\gamma}' \boldsymbol{z}_{st} + \log(\text{person-days}_{st}) \quad (1)$$

where Y_{st} is the count of CCS level 1-defined cause-specific hospitalizations in period t within stratum s; α_s are the stratum-specific intercepts; Exposure_{lst} is an indicator of whether the given period is the exposure period ($\ell = 0$) or one of the lag weeks following an exposure ($\ell \in [1, 4]$); $\boldsymbol{z}_{st}$ is the vector of confounder values; and $\log(\text{person-days}_{st})$ is the person-time offset, computed as the number of Medicare enrollees in the corresponding ZIP code and year multiplied by the length of the period in days. The β_ℓ are log hospitalization rate ratios for exposure vs control periods and their corresponding lags. The $\boldsymbol{\gamma}$ are the regression coefficients corresponding to the vector of confounders.

S.3.2 Estimation of flood-related risks

We report estimated relative percentage changes in hospitalization rates for the flood exposure period and each lag as $100 \times \left(\exp(\widehat{\beta}_\ell) - 1\right)$. We also calculated the mean (percentage) effect of flood exposure on hospitalizations over the duration of the flood and the four lag weeks. Standard errors for the corresponding confidence intervals $100(1 - \alpha)\%$ were estimated using the delta method.

S.3.3 Multiple comparisons correction

We apply Bonferroni corrections to 95% confidence intervals by using $\alpha = 0.05/13$, since we consider 13 CCS level 1 causes in our primary analysis.

S.3.4 Additional analyses

We considered longer lag periods beyond the 4-week window in order to better characterize and identify the medium-term impacts of severe flood events. The results are given in Section S.8.2.

We assessed the sensitivity of our results to the exposure criteria we established for our main analyses. We conducted analyses using a "less stringent" exposure criteria: all ZIP codes that experienced flood exposure during the study period, regardless of percentage and/or area flooded, were treated as exposed and included in the analyses. We also considered a "more stringent" exposure criteria: only ZIP codes that had at least 1 % flooded and/or 10 square-miles of surface area flooded were treated as exposed and included in the analyses. To further isolate

flood-specific effects, we fit a model on only ZIP Codes impacted by flood events caused by heavy rain (excluding tropical storms) with the same exposure definition used in the primary analysis. Lastly, we fit a model that included ZIP codes impacted by floods caused by dam faults or breakage and snowmelt/ice melt, which tend to have longer durations and may not be naturally occurring for dams, in addition to the causes considered in our primary analysis (heavy rain and tropical storms). Results are shown in Section S.8.3.

Additionally, we explored the sensitivity of our results to our approach to confounding adjustment in the main analyses. First, we conducted analyses using a different selection of matched controls. Specifically, for each ZIP code-flood exposure period, we used data from the same ZIP code and time period for *all* years during the study (rather than only the two closest years) that did not experience an exposure during the relevant time period. Second, to assess the possibility for residual confounding by location-specific long-term trends, we conducted analyses adjusting for a spline on year, climate region as designated by the U.S. National Centers for Environmental Information [5], and an interaction between the two. Third, we conducted analyses varying our choice of spline degrees of freedom for time-varying confounders such as year and meteorological factors. The results of these sensitivity analyses are shown in Section S.8.4.

S.4 Sub-cause analysis

We conducted an analysis mimicking our main analysis for each sub-cause of hospitalization (CCS level 3 causes) that had at least 20,000 hospitalizations in our study sample during the study period. This analysis is restricted to only these 59 sub-causes due to numerical instability that can result from low hospitalization counts at the ZIP code-level. To keep the results tractable, for each sub-cause we present the association between flood exposure and hospitalization rate as a mean (percentage) change in hospitalization rate over the flood exposure period and four lag weeks. These results are shown in Figure S.1. Sub-causes are categorized according to their corresponding broader cause. To represent the general trend for each of the 13 broad causes of hospitalization (CCS level 1 causes), the mean percentage changes in hospitalization rate with Bonferroni-corrected 95% confidence interval over the studied time period is shown at the top of each panel.

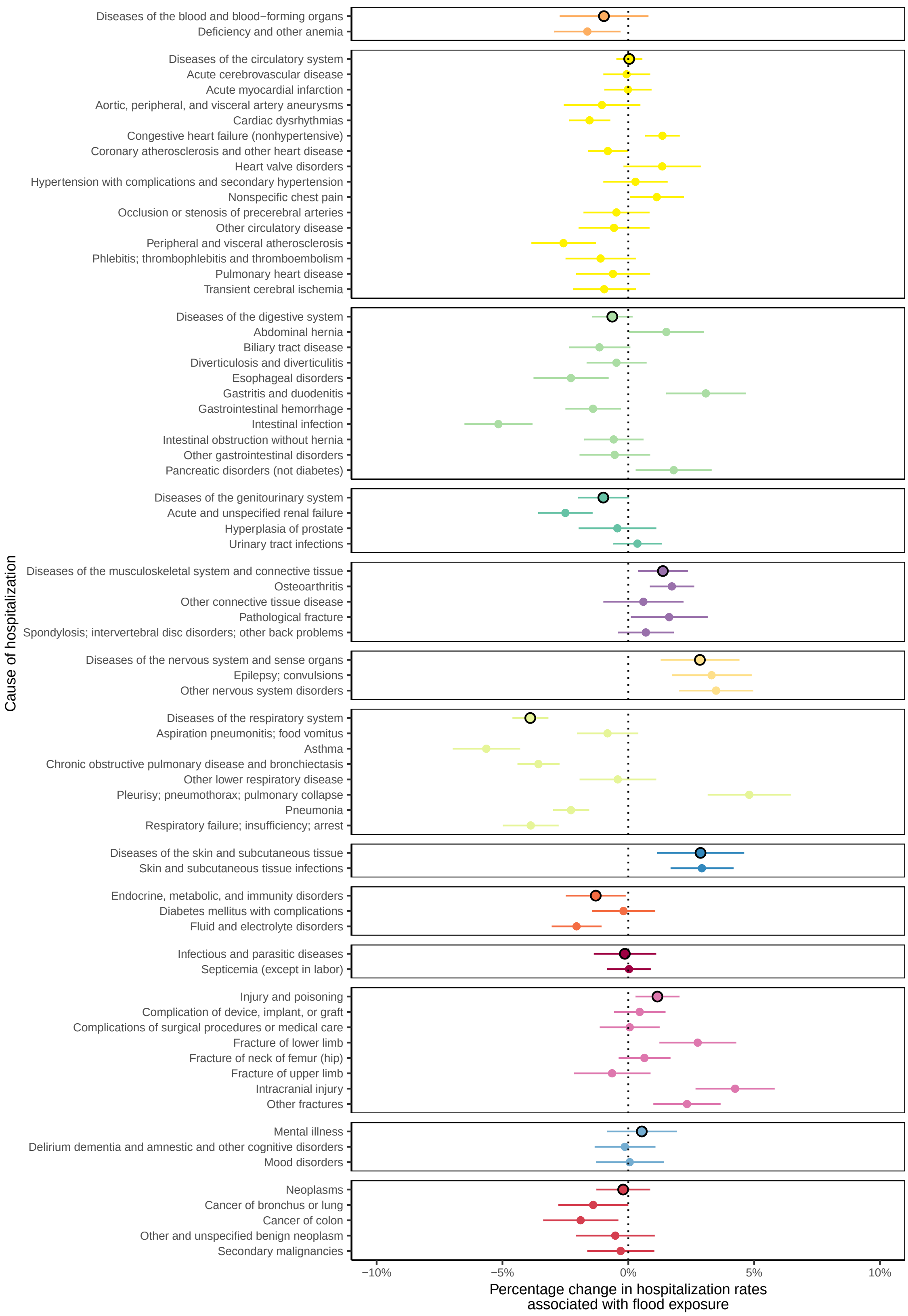

Figure S.1: **Mean percentage change in cause-specific hospitalization rates with flood exposure by sub-cause.** Average percentage change in hospitalization rates is over the flood exposure period and all four lag weeks. Dots show point estimates and error bars represent 95% confidence intervals.

S.5 Stratified analysis by season

In Figure S.2, we investigated the association between flood exposure and hospitalization rates by season. We categorized flood events that started between May and October as warm season events and those that started in November through April as cold season events. We observed that flood events occurring in the warm season largely drove increased hospitalization rates for causes we noted as consistently significant in our primary analysis. Specifically, these causes were nervous system diseases, injuries and poisoning, and skin diseases. Increases in musculoskeletal system diseases (in lag weeks 3-4) were driven by flood events in the cold season. Notably, decreases in respiratory system hospitalizations were driven by flood events during the cold season while we see consistent evidence of increased respiratory-related hospitalization rates for flood events occurring during the warm season. A similar pattern emerged for infectious and parasitic diseases as well as circulatory system diseases (during flood exposure and lag weeks 1-2).

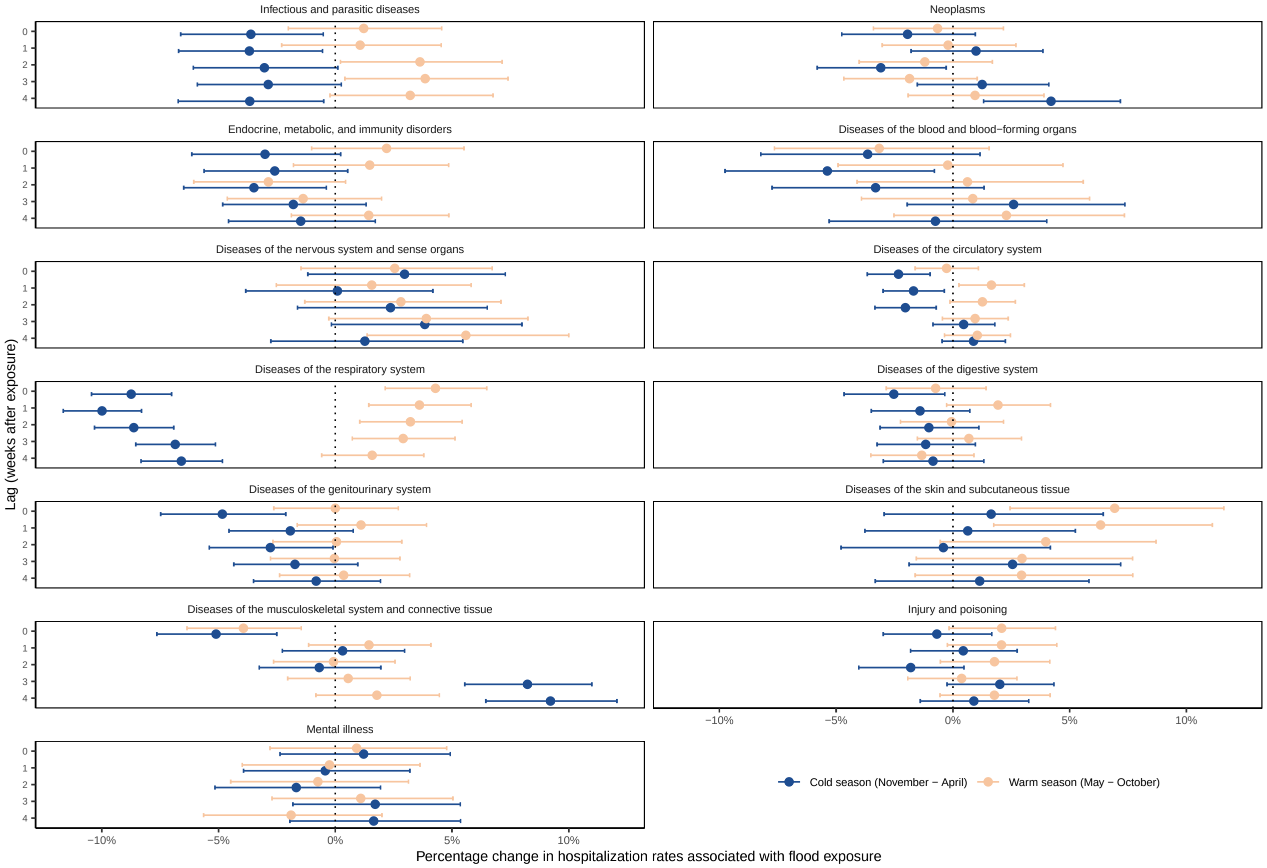

Figure S.2: **Season-stratified percentage changes in cause-specific hospitalization rates during and after flood exposure by cause and lag time.** Dots show point estimates and error bars represent Bonferroni-corrected 95% confidence intervals.

S.6 Stratified analysis by admission type

We also examined the association between flood exposure and hospitalization rates by type of hospital admission (emergency vs non-emergency) as shown in Figure S.3. Generally, non-emergency hospitalization rates decreased or showed no significant change during the flood (lag 0) except for nervous system diseases, mental illness, and skin and subcutaneous tissue diseases. For the aforementioned causes, we saw increases in non-emergency hospitalizations for multiple lag weeks. Emergency hospitalizations increased for the exposure period and all lag weeks for nervous system diseases, injury-related hospitalizations, and skin and subcutaneous tissue diseases. Conversely, for respiratory system diseases, we observed decreases across all lag periods for emergency hospitalizations and non-emergency hospitalizations (except lag 3 for the latter). For other causes, there tended to be little to no changes for emergency hospitalizations. Due to low case counts for infectious and parasitic non-emergency hospitalizations, we were unable to achieve model convergence.

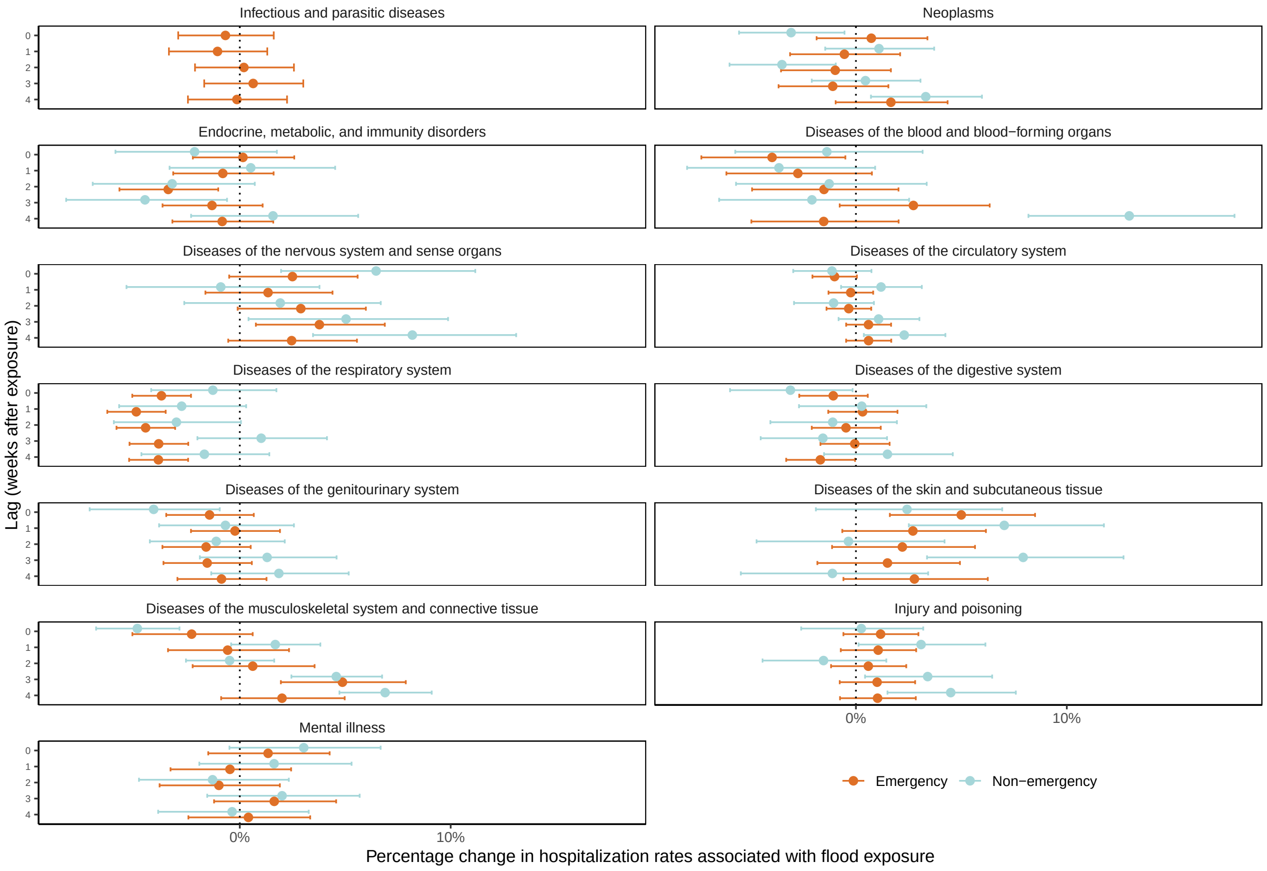

Figure S.3: **Admission type-stratified percentage changes in cause-specific hospitalization rates during and after flood exposure by cause and lag time.** Dots show point estimates and error bars represent Bonferroni-corrected 95% confidence intervals.

S.7 Stratified analyses by socioeconomic status indicators

To understand additional drivers and dimensions of vulnerability beyond proportion of Black residents, we conducted stratified analyses by ZIP code-level (1) median household income and (2) poverty rate. These results are shown in Figures S.4 and S.5, respectively. Generally, for ZIP codes with lower median household incomes, rates of hospitalization for skin diseases, nervous system diseases, injuries, musculoskeletal system diseases (lag weeks), mental health-related impacts (during flood exposure only) were higher than than that of ZIP codes with higher median household incomes. There were no notable differences in hospitalization rates for other disease causes between household income-level across the flood exposure period and lag weeks. In ZIP codes with higher poverty rates, there were decreased rates of hospitalization for respiratory system diseases and increased rates of hospitalization for injuries and skin diseases, compared to ZIP codes with lower poverty rates, during the exposure period and lag weeks. We also observed increased rates of hospitalization for mental health-related impacts and nervous system impacts during flood exposure for communities with higher rates of poverty. Other causes of hospitalization revealed little to no changes with no distinct patterns between ZIP codes with different levels of poverty.

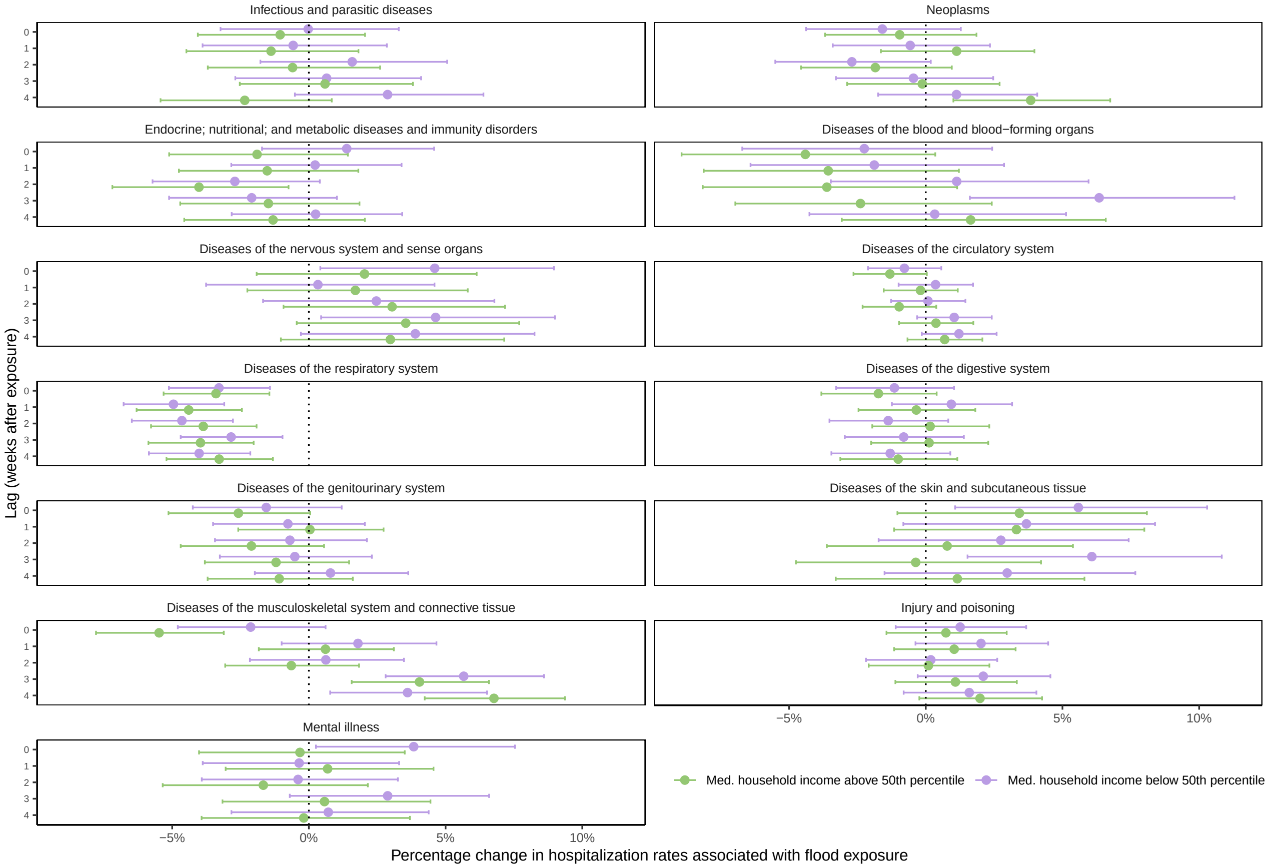

Figure S.4: **Percentage changes in cause-specific hospitalization rates during and after flood exposure by cause and lag time, stratified by ZIP code-level median household income.** Dots show point estimates and error bars represent Bonferroni-corrected 95% confidence intervals.

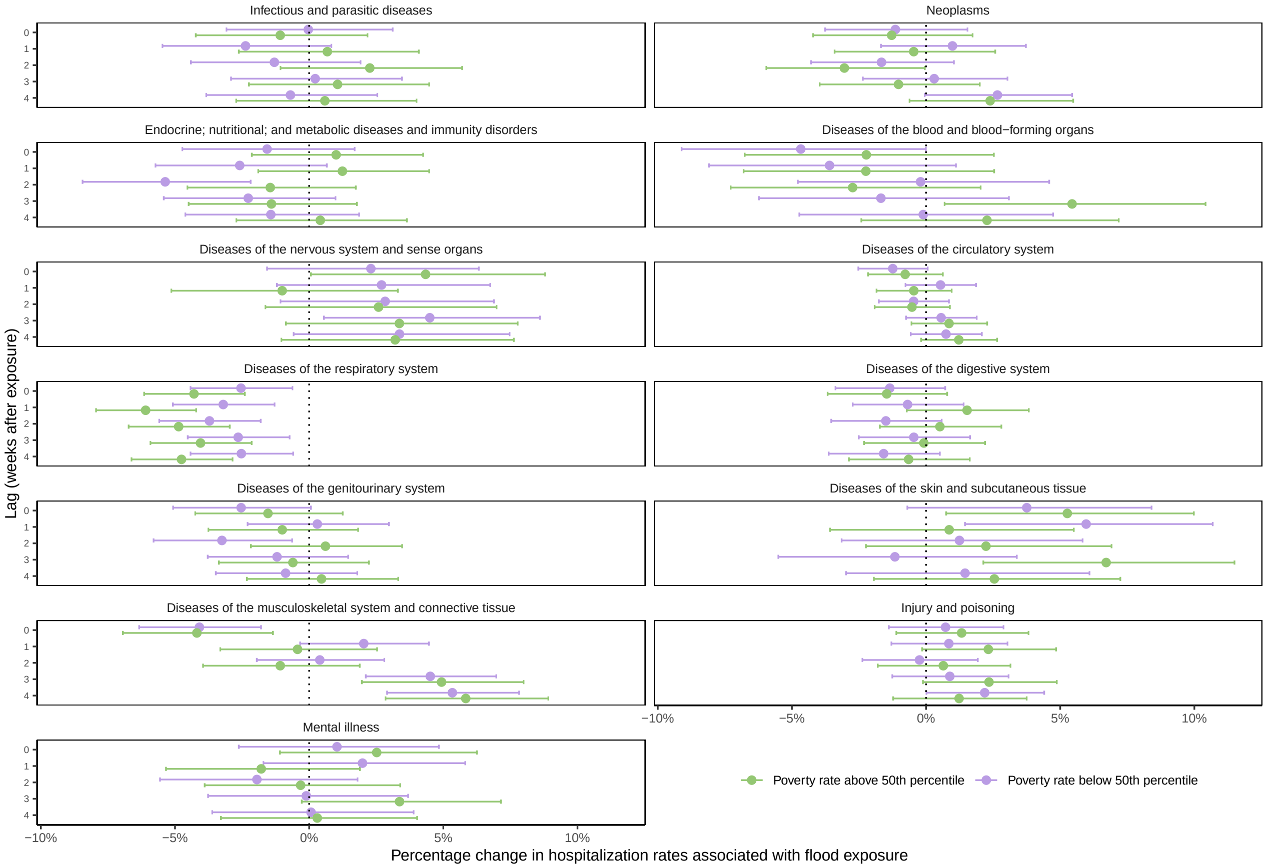

Figure S.5: **Percentage changes in cause-specific hospitalization rates during and after flood exposure by cause and lag time, stratified by ZIP code-level poverty rate.** Dots show point estimates and error bars represent Bonferroni-corrected 95% confidence intervals.

S.8 Additional analyses

S.8.1 Descriptive plots

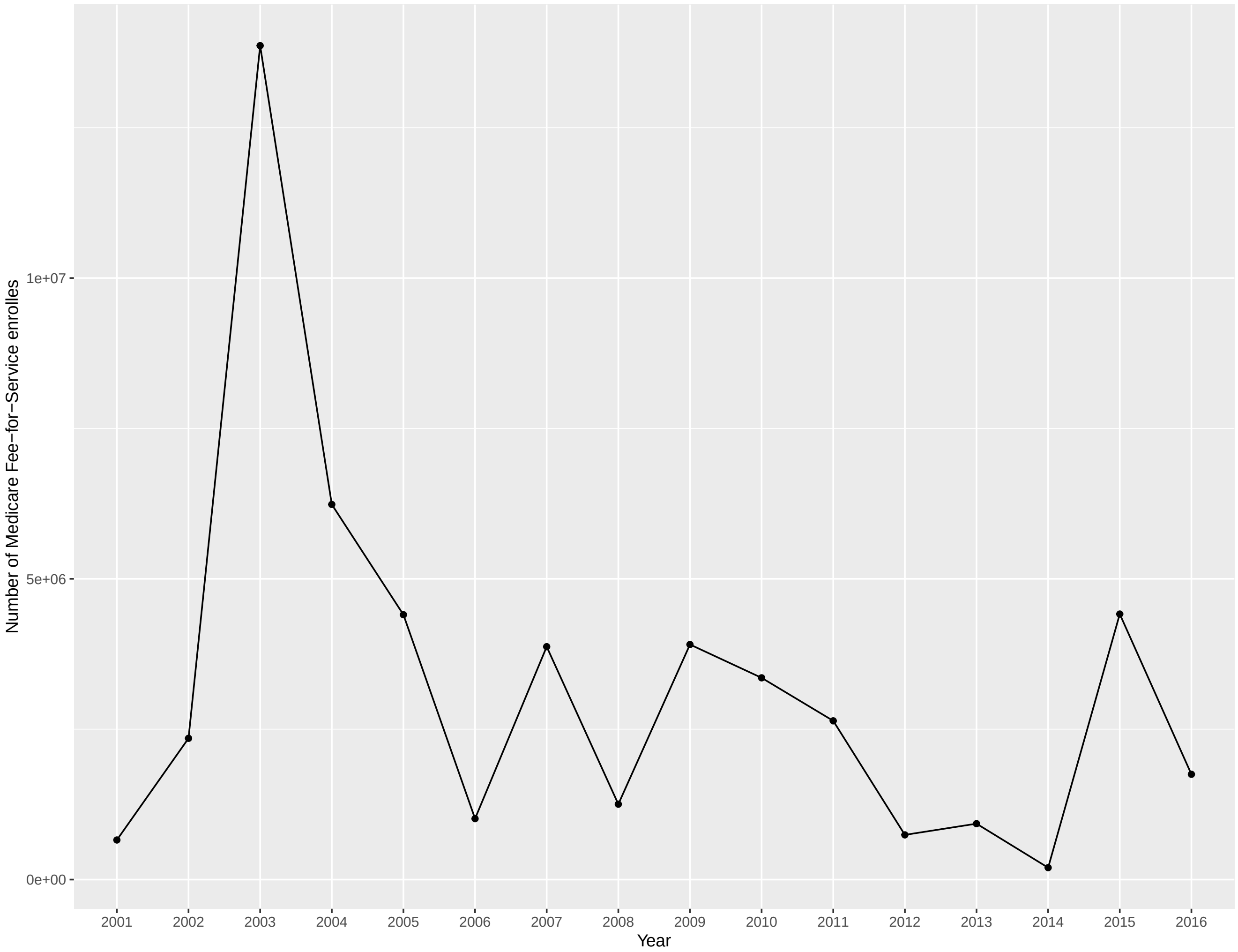

Figure S.6: Number of Medicare fee-for-service enrollees living in ZIP Codes with at least one flood exposure in 2000-2016.

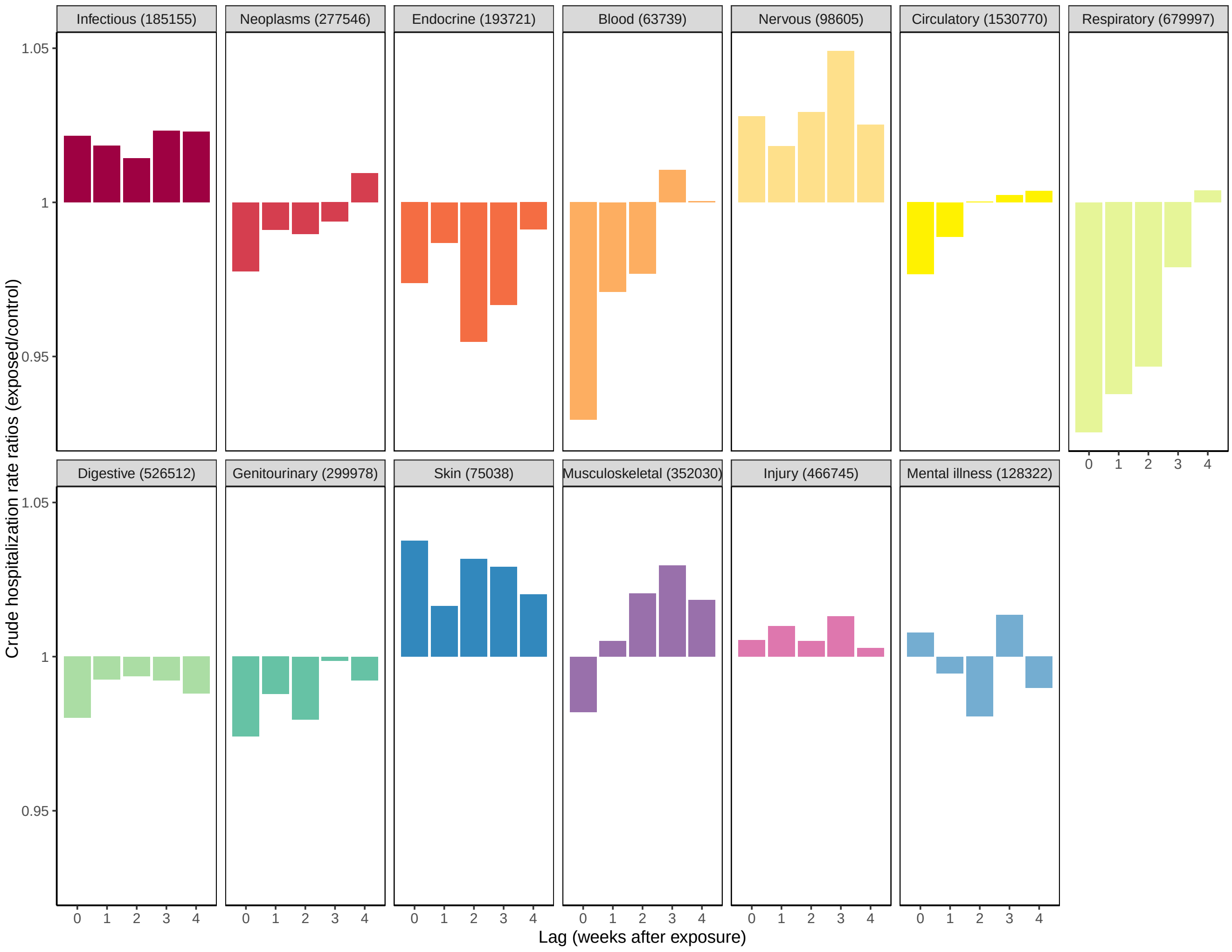

Figure S.7: **Crude hospitalization rate ratios (comparing flood exposure to matched controls) by cause and lag time for ZIP codes with at least one flood exposure in 2000-2016.** Each panel heading lists the cause of hospitalization and the observed number of hospitalizations in our analytic dataset for that cause.

S.8.2 Evaluation of longer lag periods to further characterize medium-term impacts

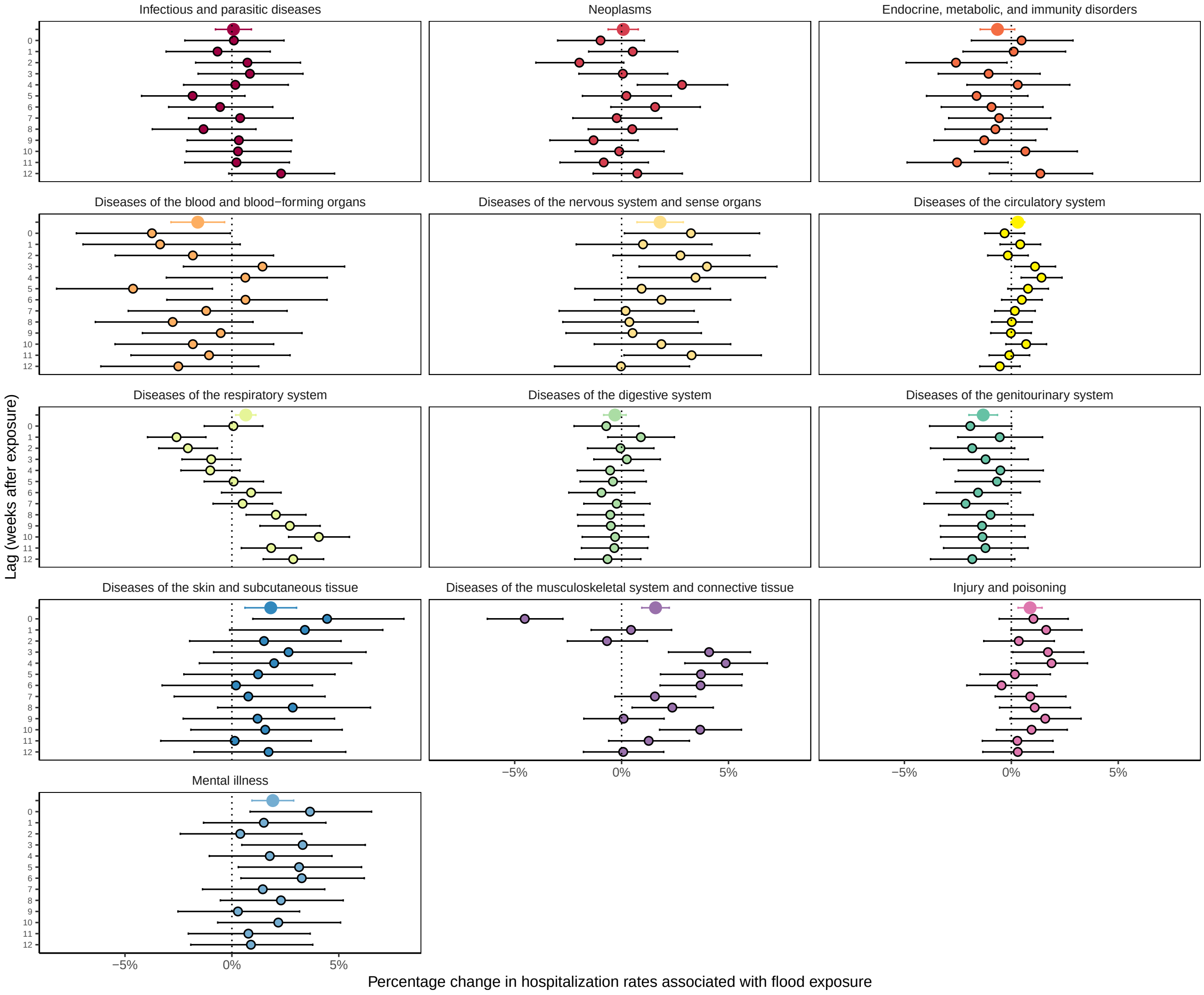

Figure S.8: **Percentage changes in cause-specific hospitalization rates during and after flood exposure by cause and extended lag time up to 12 weeks.** Dots show point estimates and error bars represent Bonferroni-corrected 95% confidence intervals. The mean percentage change in hospitalization (with corrected 95% confidence interval) over the flood exposure period and all 12 lag weeks is shown at the top of each panel.

S.8.3 Sensitivity analyses: alternative flood exposure definitions

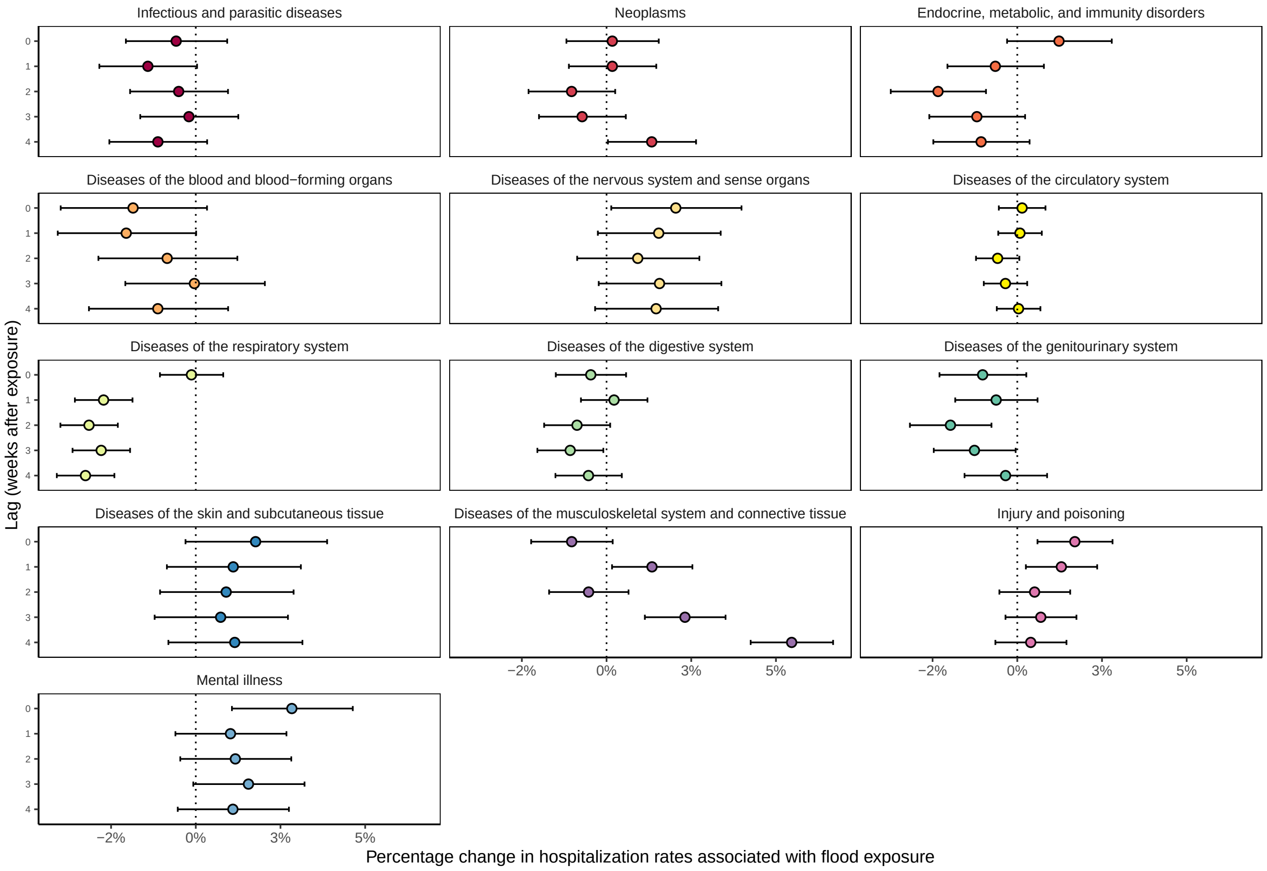

Figure S.9: **Percentage changes in cause-specific hospitalization rates during and after flood exposure by cause and lag time for floods caused by heavy rain, tropical storms, ice/snowmelt, or dam faults with any level of exposure.** Dots show point estimates and error bars represent Bonferroni-corrected 95% confidence intervals.

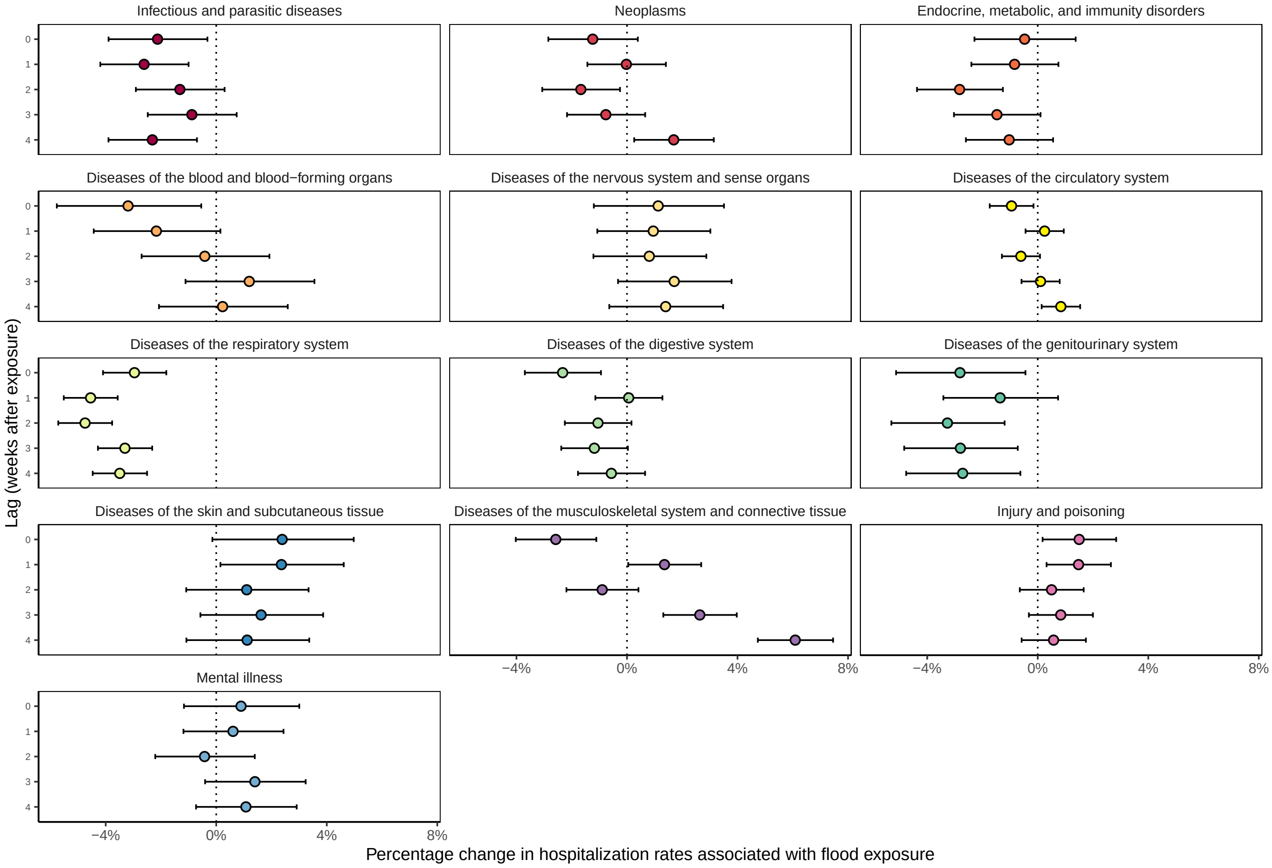

Figure S.10: **Percentage changes in cause-specific hospitalization rates during and after flood exposure by cause and lag time for floods caused by heavy rain or tropical storms with any level of exposure.** Dots show point estimates and error bars represent Bonferroni-corrected 95% confidence intervals.

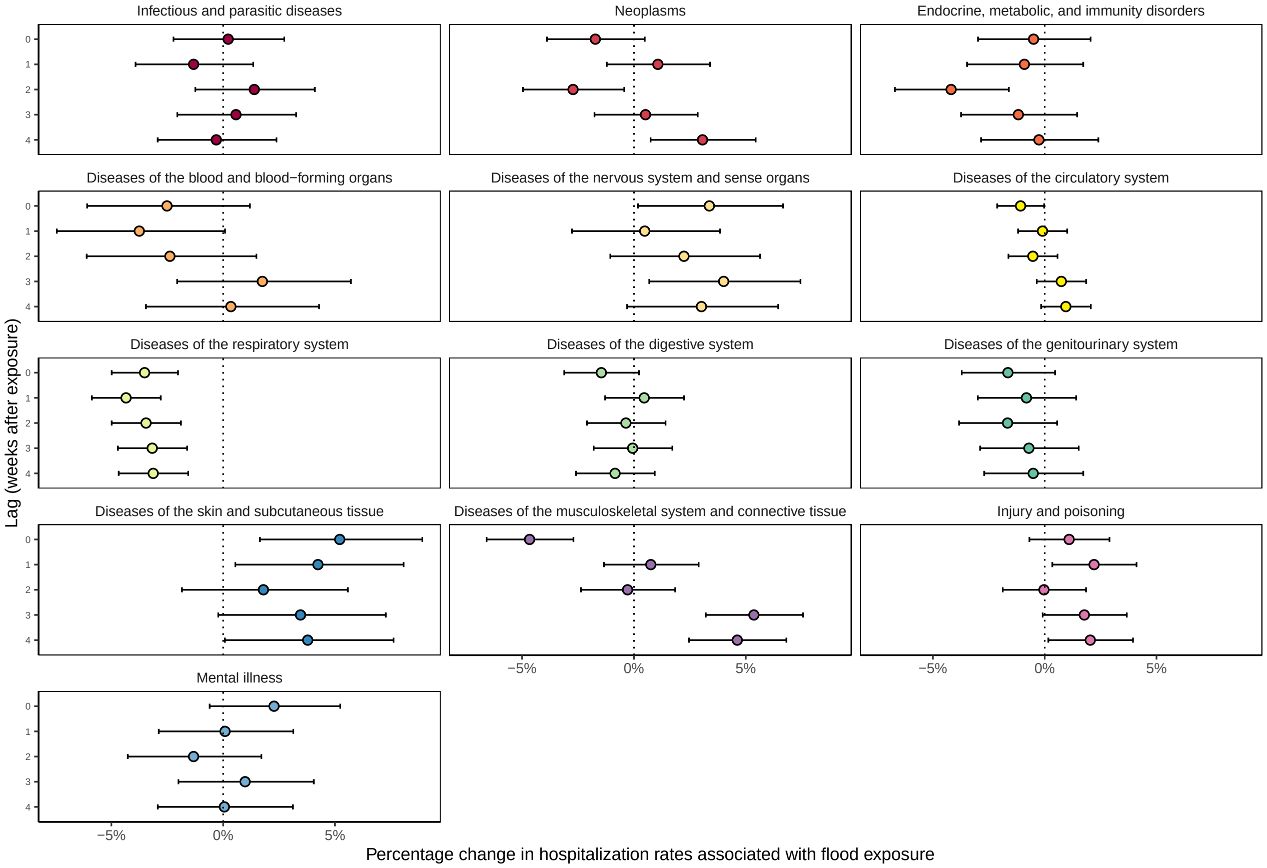

Figure S.11: **Percentage changes in cause-specific hospitalization rates during and after flood exposure by cause and lag time for floods caused by heavy rain or tropical storms with at least 1% flooded and/or a flooded area of at least 10 square-miles.** Dots show point estimates and error bars represent Bonferroni-corrected 95% confidence intervals.

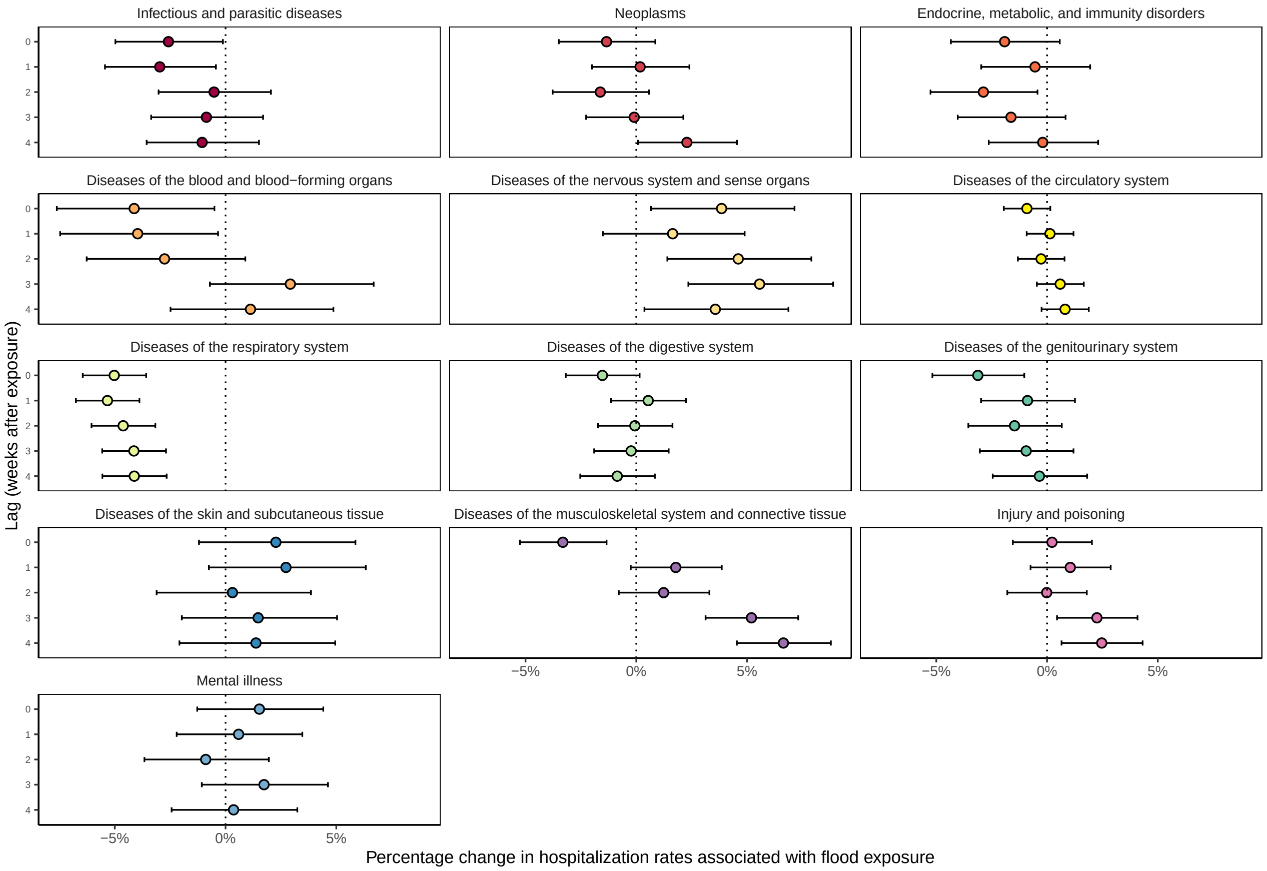

Figure S.12: **Percentage changes in cause-specific hospitalization rates during and after flood exposure by cause and lag time for floods caused by heavy rain with at least 0.5% flooded and/or a flooded area of at least 5 square-miles** Dots show point estimates and error bars represent Bonferroni-corrected 95% confidence intervals.

S.8.4 Sensitivity analyses: residual confounding

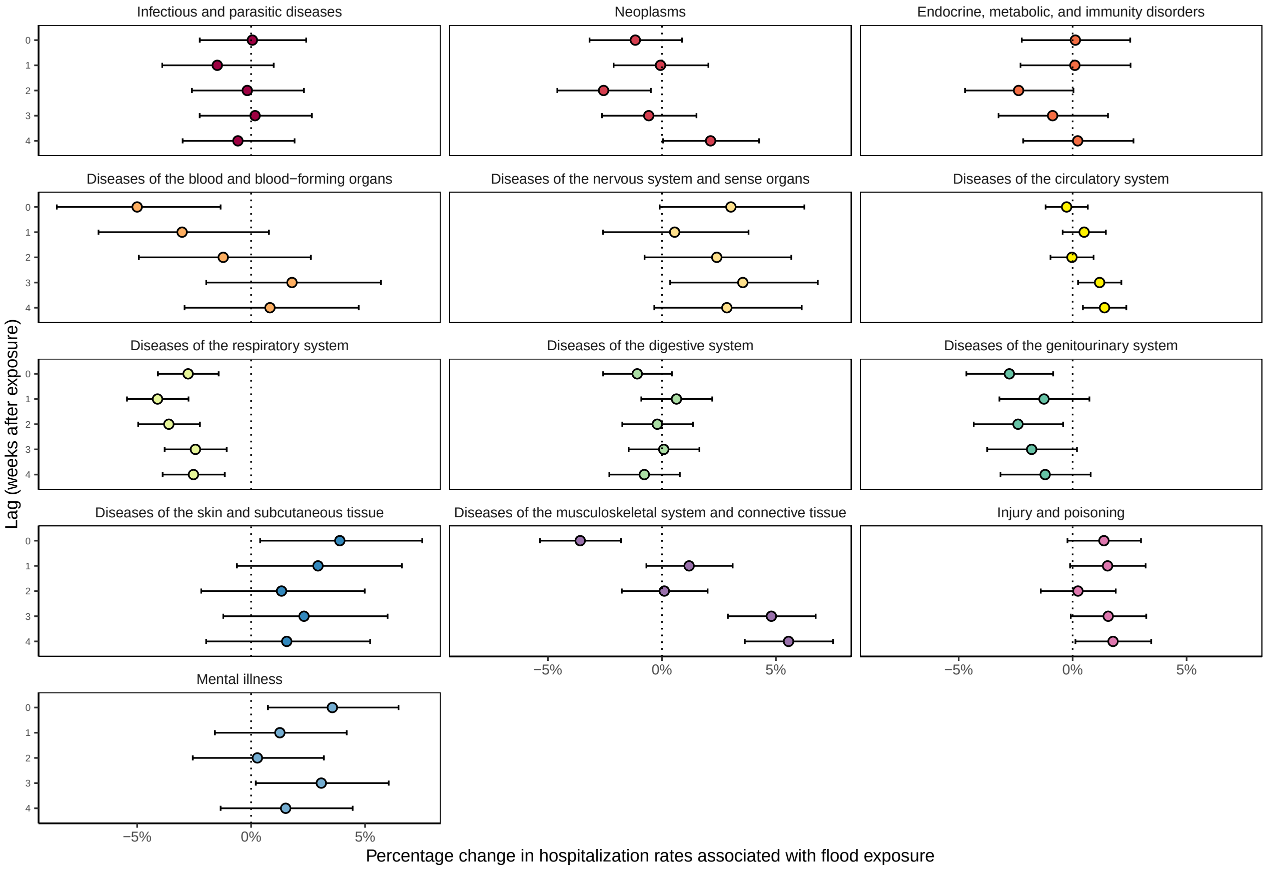

Figure S.13: **Percentage changes in cause-specific hospitalization rates during and after flood exposure by cause and lag time, from analyses using all matched control years.** Dots show point estimates and error bars represent Bonferroni-corrected 95% confidence intervals.

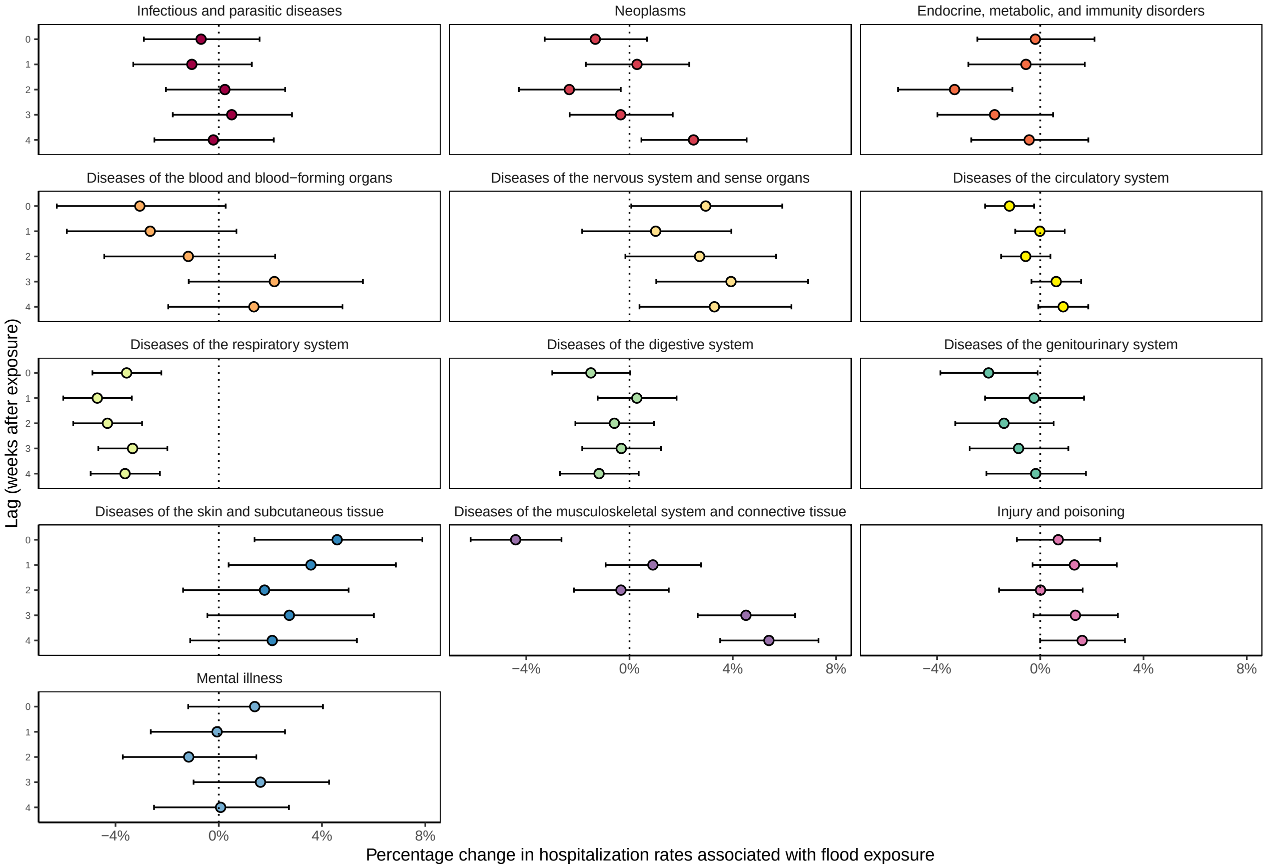

Figure S.14: **Percentage changes in cause-specific hospitalization rates during and after flood exposure by cause and lag time, from analyses incorporating model-based adjustment of location-specific long-term trends.** In addition to long-term trends in hospitalization patterns and daily meteorology, we include an interaction term between year (via a spline) and climate region. Dots show point estimates and error bars represent Bonferroni-corrected 95% confidence intervals.

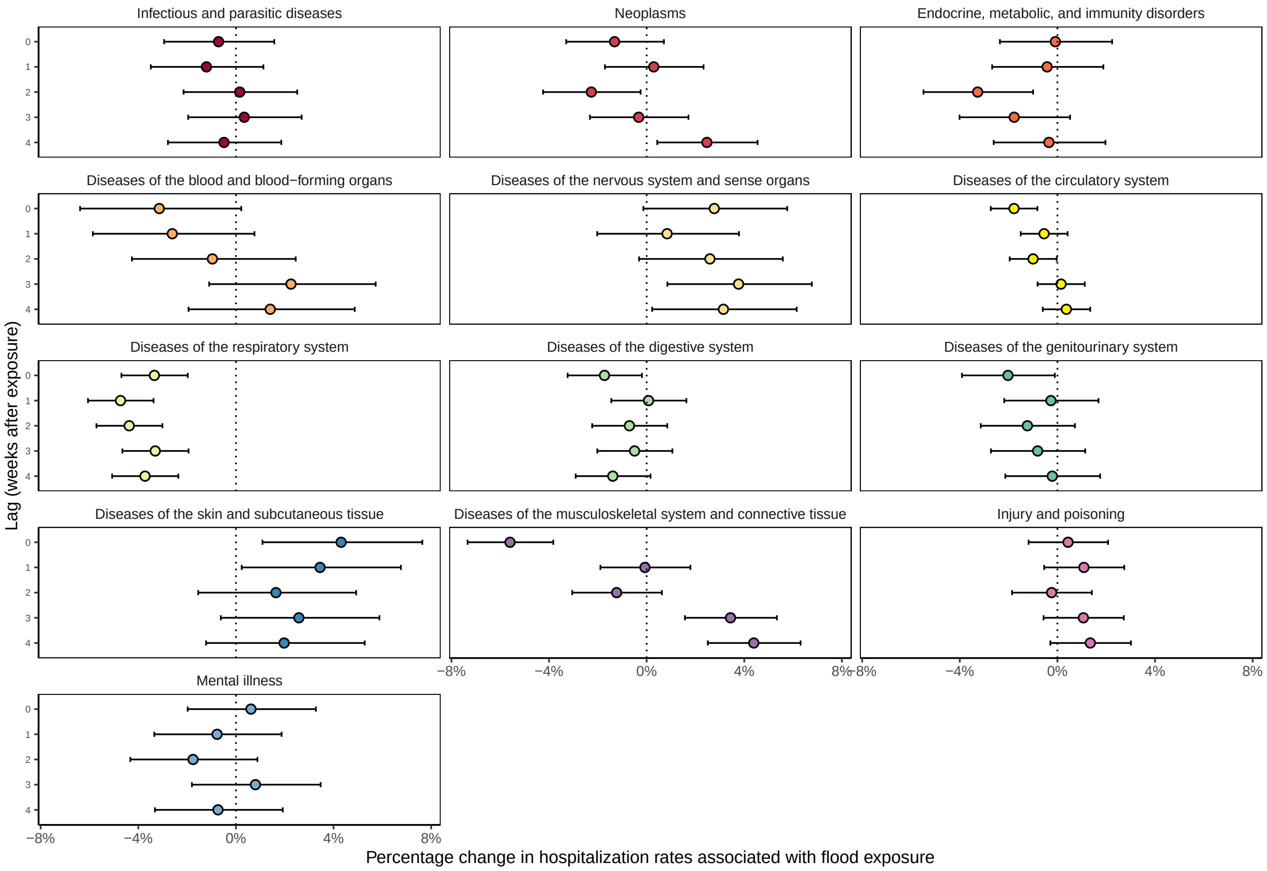

Figure S.15: **Percentage changes in cause-specific hospitalization rates during and after flood exposure by cause and lag time, from analyses adjusting for time-varying confounders with natural splines with three degrees of freedom.** Dots show point estimates and error bars represent Bonferroni-corrected 95% confidence intervals.

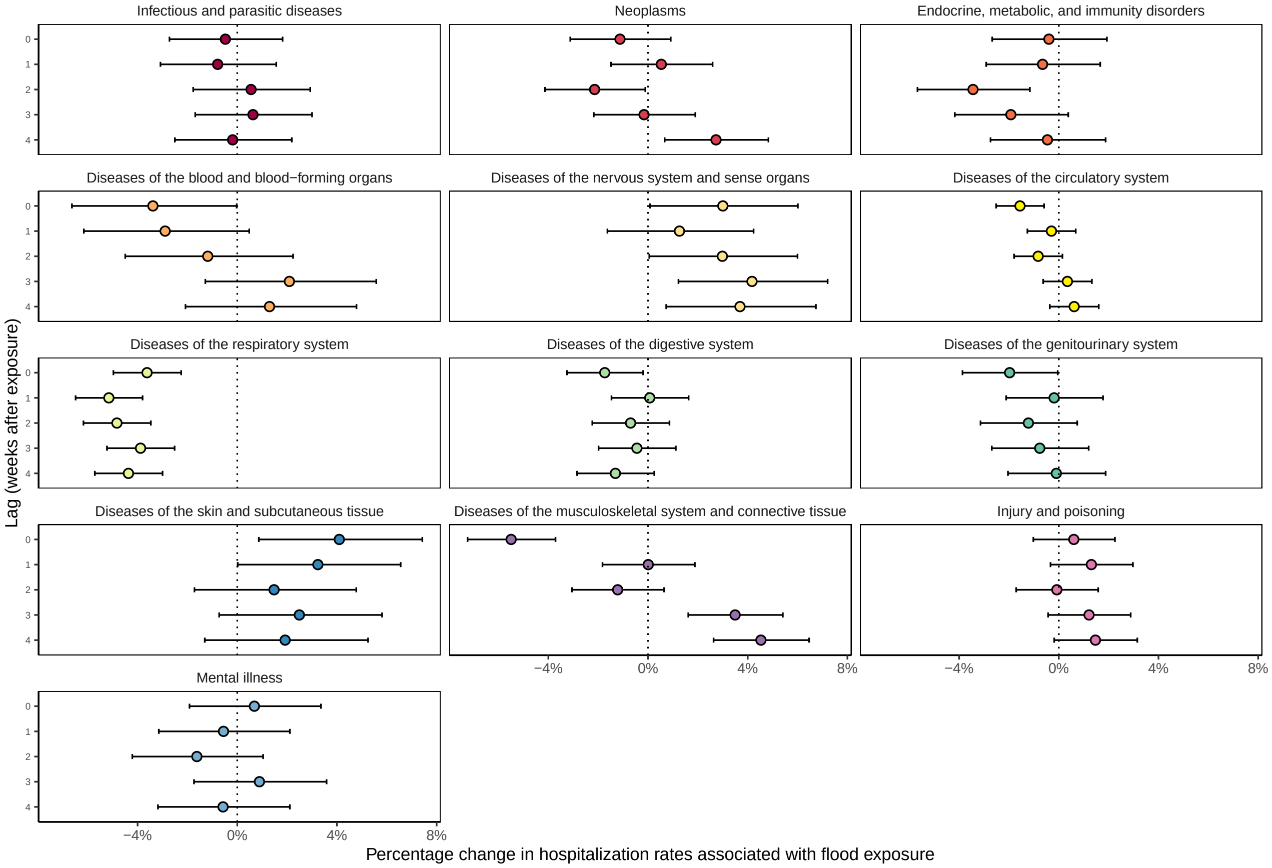

Figure S.16: **Percentage changes in cause-specific hospitalization rates during and after flood exposure by cause and lag time, from analyses adjusting for time-varying confounders with natural splines with four degrees of freedom.** Dots show point estimates and error bars represent Bonferroni-corrected 95% confidence intervals.

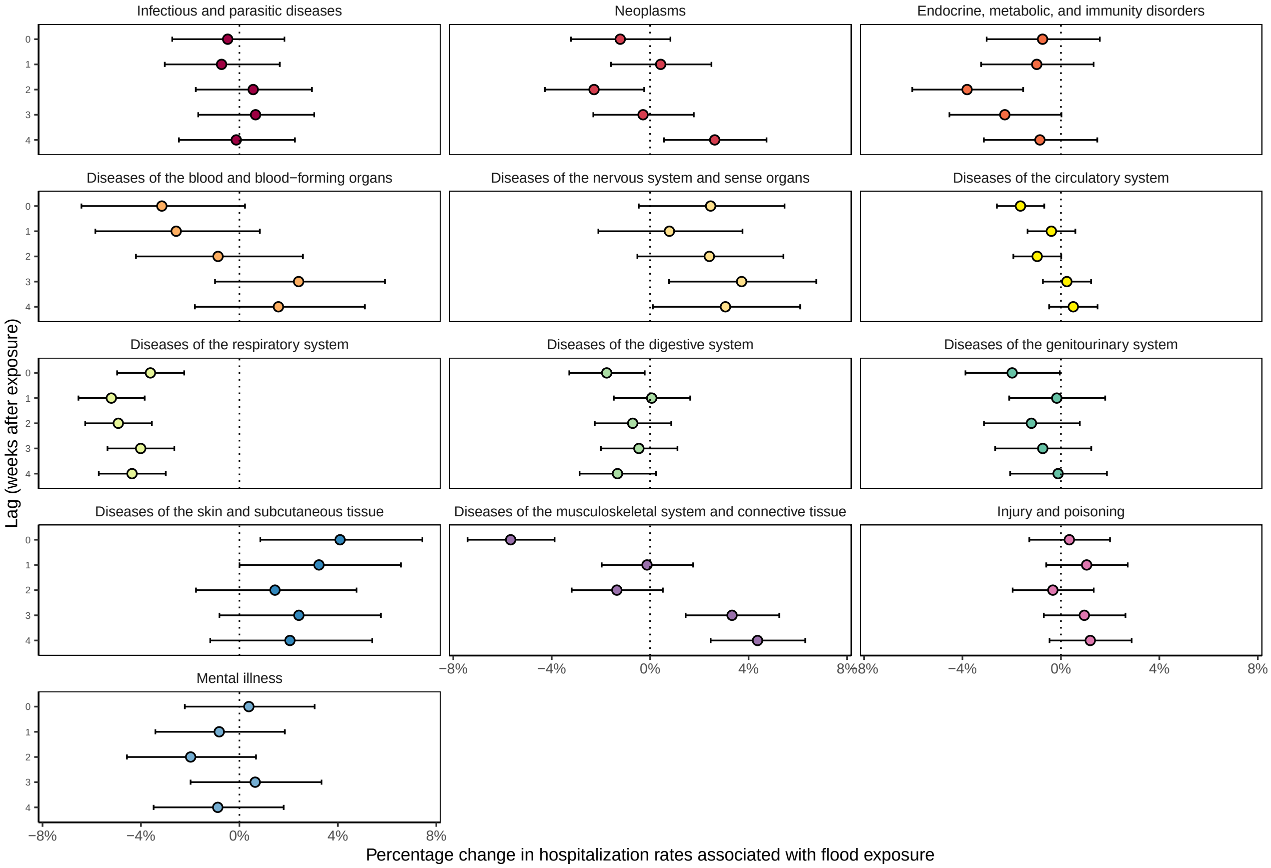

Figure S.17: **Percentage changes in cause-specific hospitalization rates during and after flood exposure by cause and lag time, from analyses adjusting for time-varying confounders with natural splines with five degrees of freedom.** Dots show point estimates and error bars represent Bonferroni-corrected 95% confidence intervals.

References

[1] B. Tellman et al. "Satellite imaging reveals increased proportion of population exposed to floods". en. In: *Nature* 596.7870 (Aug. 2021), pp. 80–86. ISSN: 0028-0836, 1476-4687. URL: https://www.nature.com/articles/s41586-021-03695-w.

[2] G.R. Brakenridge. *Global Active Archive of Large Flood Events*. 2023. URL: https://floodobservatory.colorado.edu/Archives/.

[3] CRED. *Em-Dat: the International Disaster Database*. 2023. URL: https://public.emdat.be/.

[4] *MODIS (Moderate Resolution Imaging Spectrometer)*. 2014. URL: https://modis.gsfc.nasa.gov/about/.

[5] Thomas Karl and Walter James Koss. "Regional and national monthly, seasonal, and annual temperature weighted by area, 1895-1983". In: (1984).
